# Biosynthetic gene clusters in *Pseudomonas viridiflava* have a fitness cost during *Arabidopsis thaliana* infection

**DOI:** 10.1128/msystems.00212-26

**Published:** 2026-06-15

**Authors:** Alejandra Duque-Jaramillo, Efthymia Symeonidi, Manuela Neumann, Haim Ashkenazy, Madelyn Allen, Detlef Weigel, Talia L. Karasov

**Affiliations:** 1 Department of Molecular Biology, Max Planck Institute for Biology Tübingen 551907, Tübingen, Germany; 2 School of Biological Sciences, University of Utah 172838, Salt Lake City, Utah, USA; 3 Institute for Bioinformatics and Medical Informatics, University Tübingen, Tübingen, Germany; Universiteit Leiden, Leiden, Netherlands

**Keywords:** *Pseudomonas*, *Arabidopsis thaliana*, biosynthetic gene clusters, specialized metabolites, fitness, infection

## Abstract

Specialized or secondary metabolites mediate biotic interactions, including virulence and defense. In plant-pathogenic *Pseudomonas*, certain specialized metabolites can enhance colonization of plant hosts, yet their broader contribution to plant–microbe interactions and the relative importance of different metabolites remain unclear. Specialized metabolites are products of enzymes encoded in biosynthetic gene clusters (BGCs), whose prediction from genome sequences has become routine but whose functional roles are rarely tested experimentally. Here, we characterize the BGC repertoire of 225 *P. viridiflava* isolates from *Arabidopsis thaliana* and assess BGC contributions to fitness and disease severity *in planta*. The BGC landscape of *P. viridiflava* was dominated by non-ribosomal peptide synthetase (NRPS) and NRPS-like BGCs, which accounted for 50% of the predicted BGCs. One-third of the BGC families were restricted to a single isolate. Transposon mutagenesis coupled with random barcode transposon sequencing (RB-TnSeq) revealed that the majority of BGCs reduce rather than increase fitness during *A. thaliana* infection, with the magnitude of the fitness cost varying across host genotypes. This cost could be due to exploitation of public goods by cheater mutant strains. In single-isolate plant infections, where public goods are not available, we found 11/34 BGC families correlated with disease severity. Yet, only two of these (an N-acetylglutaminylglutamine amide [NAGGN] and an NRPS) were negatively associated with disease severity, which is positively correlated with bacterial growth in this pathosystem, further indicating that BGCs are generally not beneficial *in planta*. Our findings reveal extensive and largely uncharacterized biosynthetic potential in populations of *P. viridiflava* and indicate that candidate metabolites are likely not adaptive for direct interactions with the plant, but perhaps for microbe-microbe interactions either *in planta* or in other ecological niches.

**IMPORTANCE:**

Bacteria, including plant-associated bacteria such as *Pseudomonas viridiflava*, produce a vast array of chemical compounds, called secondary or specialized metabolites, that can mediate their interaction with the plant host or other microorganisms. Some of these compounds are known to directly influence how bacteria interact with plants, but it has been unclear whether this is a general rule. We studied a large collection of closely related leaf-dwelling *P. viridiflava*—a plant pathogen—that varied in their ability to cause disease. We found that very few of the gene clusters responsible for making specialized metabolites improved the ability of the bacteria to colonize its natural host *Arabidopsis thaliana*. On the contrary, carrying these gene clusters often reduced bacterial growth and disease severity in plants. Specialized metabolites may instead primarily be important for interacting with other microbes, different host species, or under environmental conditions we did not test. These are questions that remain for future research.

## INTRODUCTION

Bacteria can produce a wide range of specialized metabolites, also known as secondary metabolites, which mediate interactions with other organisms and generally help bacteria to cope with their environment. Specialized metabolites are defined as metabolites that are not essential for basal bacterial growth or division but that instead play a role in nutrient acquisition, quorum sensing, defense, and virulence (1). Specialized metabolites also mediate the interaction between bacteria and the host organisms they colonize, such as plants (2). Some of these metabolites are well known to affect plant growth and plant defense against pathogenic bacteria. Examples of specialized metabolites that mediate plant-bacterial interactions and contribute to the virulence of plant-pathogenic *Pseudomonas* species include toxins such as coronatine, syringomycin, and phaseolotoxin (3); the iron-chelating siderophore pyoverdin (4); and biosurfactants syringafactin and cichofactin, which increase the availability of water-insoluble substrates and promote swarming (5, 6).

Specialized metabolites are produced by proteins encoded in biosynthetic gene clusters (BGCs). A BGC is a consecutive array of at least two genes that encode the biosynthetic pathway for the production of these metabolites and their variants, including biosynthetic, regulatory, and transport proteins (7, 8). BGCs often have defined patterns of different types of genes depending on the chemical class of specialized metabolites they produce; hence, they can be predicted from genome sequences. The gold-standard tool for BGC prediction in bacterial genomes is antiSMASH (antibiotics and secondary metabolite analysis shell) (9, 10), which identifies BGCs based on profile Hidden Markov Models (HMMs) of genes that are specific for each metabolite class. Genome mining for BGCs in small and large bacterial genomes data sets is now common, particularly focusing on a genus or species (11, 12) or on a particular environment, from the human microbiota (13) to oceans (14), soil (15, 16), and many other habitats (17, 18).

*Pseudomonas viridiflava* is a globally distributed agricultural pest (19–21) and a natural pathogen of *Arabidopsis thaliana* (22–24), but its mechanisms of virulence are largely unknown. *Pseudomonas viridiflava* is prevalent in the epi- and endophytic leaf compartments in *A. thaliana* populations in Southwest Germany and across Europe (24, 25). Most isolates are pathogenic on *A. thaliana* under lab conditions, with inter-strain variation in virulence (21, 24, 26, 27). These *P. viridiflava* isolates, referred to as ATUE5 as they were first isolated around Tuebingen, encode two known virulence factors: the effector AvrE and the pectate lyase gene *pel*, and lack other well-known effectors proteins and toxins (24).

The *Pseudomonas* genus is well known for its ability to produce a vast array of specialized metabolites (28–30), and it is one of the most biosynthetically diverse bacterial genera, second only to *Streptomyces* (31). There is great diversity in the specialized metabolite potential of different *Pseudomonas* species (29, 30, 32); yet, variation among isolates of the same species has rarely been studied. Descriptions of the BGC repertoire of plant-associated *Pseudomonas* have been published in recent years, focusing on isolates from the soil and the plant rhizosphere (12, 30, 33, 34). However, most studies did not test the importance of these BGCs in the interaction with the host and/or other microbes (35–37), particularly beyond well-known phytotoxins. Considering that *P. viridiflava* isolates are pathogenic to varying degrees on *A. thaliana* but lack effector proteins and toxins encoded by other plant-pathogenic *Pseudomonas* species, we investigated whether the specialized metabolites encoded by *A. thaliana*-associated *P. viridiflava* are involved in infection and disease severity on this host.

Here, we describe the largely uncharacterized specialized metabolite potential of 225 closely related yet genetically distinct *P. viridiflava* ATUE5 isolates previously obtained from *A. thaliana* populations in southwestern Germany (24). We not only predict the BGC repertoire from whole-genome sequences but also show that BGC-associated genes reduce fitness during plant infection, with varying magnitude between host genotypes. By integrating the pattern of BGC presence/absence with disease severity data of single-isolate infections of *A. thaliana*, we identify BGCs that negatively correlate with disease severity on plants, further corroborating that they incur fitness costs *in planta*.

## RESULTS

### The biosynthetic potential of *A. thaliana*-associated *P. viridiflava* is broad and its products are largely unknown

We selected representative *P. viridiflava* ATUE5 genomes from a published collection (24) based on gene content similarity (38). Starting with 1,338 complete genome assemblies (BUSCO single genes > 95%), we reduced our data set to 163 ATUE5 genomes with distinct gene content according to the presence and absence of groups of orthologous genes. In addition, we included the genomes of 62 ATUE5 isolates for which disease severity data were available, measured as plant size (green pixels) after infection (27). A total of 225 ATUE5 genomes were included in downstream analyses. These genomes had a median completeness of 98.1% (min = 92.1%, max = 99.2%) and 88 contigs (min = 19, max = 5,666) (Table S1). Completeness was not available for four genomes, one of which also lacked the number of contigs in the assembly. Representative genomes had higher completeness (>94%) and smaller number of contigs than genomes with disease severity data (Fig. S1).

**Fig 1:**
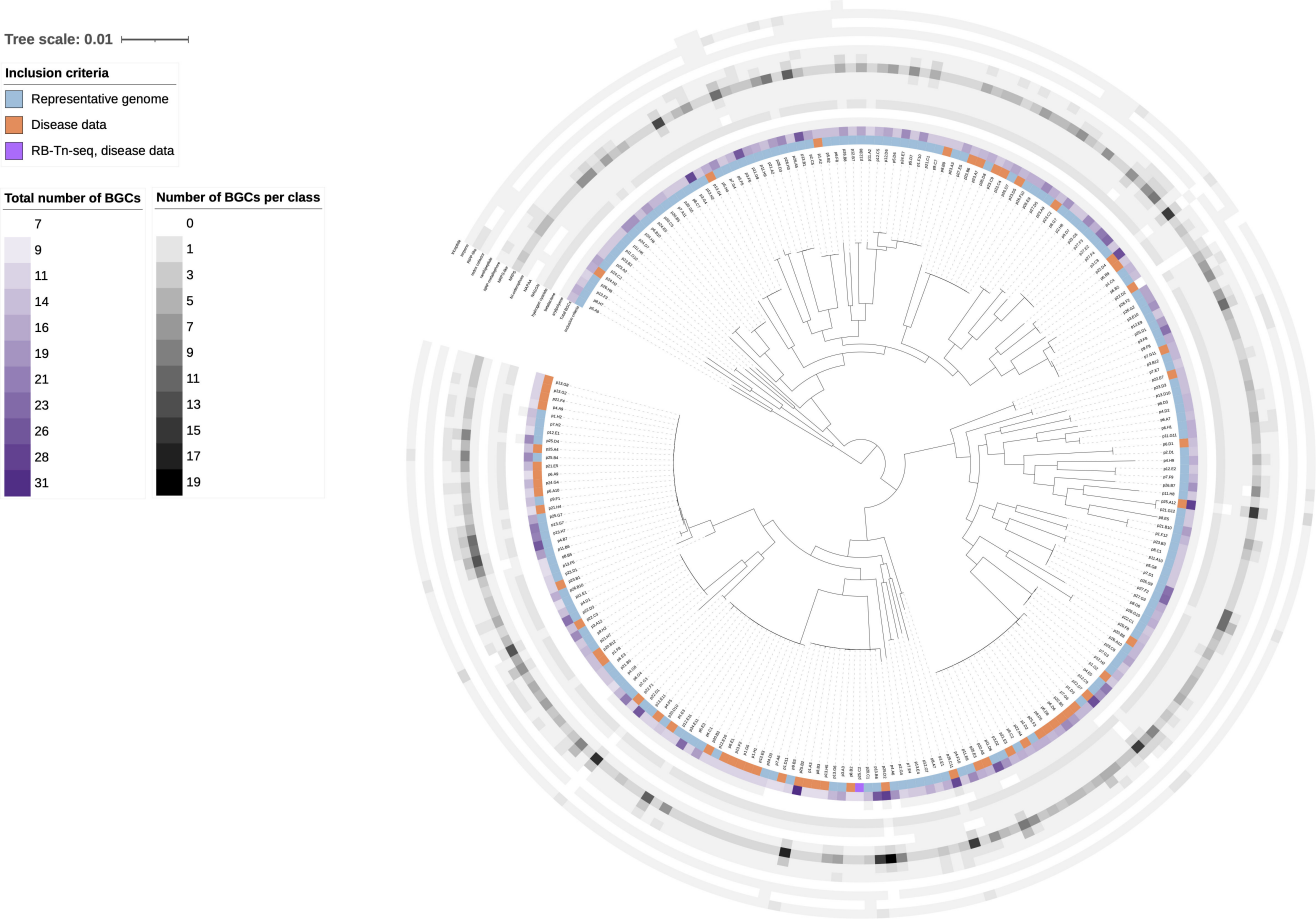
Distribution of biosynthetic gene clusters across 225 genomes of plant-associated *Pseudomonas viridiflava*. Core genome maximum likelihood phylogeny (24) of 225 *P. viridiflava* isolates from southwest Germany, built with 939 concatenated core genes. Ring color indicates the following, from innermost out: first ring: the inclusion criteria for each genome, i.e., if it was identified as a representative genome (blue) or if it was included because disease data were available (orange); purple indicates p25.C2 for which disease data were available and served as background for the Bar-Seq mutant pool. Second ring (purple): total number of predicted BGCs, outer rings (gray): number of BGCs per class, for classes arylpolyene, betalactone, hydrogen cyanide, N-acetylglutaminylglutamine amide (NAGGN), non-alpha poly-amino acids like e-polylysin (NAPAA), NRP-independent (NI) siderophore, NRPS, NRPS-like, NRP metallophore, ranthipeptide, redox cofactor, RiPP-like, terpene and triceptide. BGC: biosynthetic gene cluster; NRPS: non-ribosomal peptide synthetase; RiPP: ribosomally synthesized and post-translationally modified peptide.

**Fig 2:**
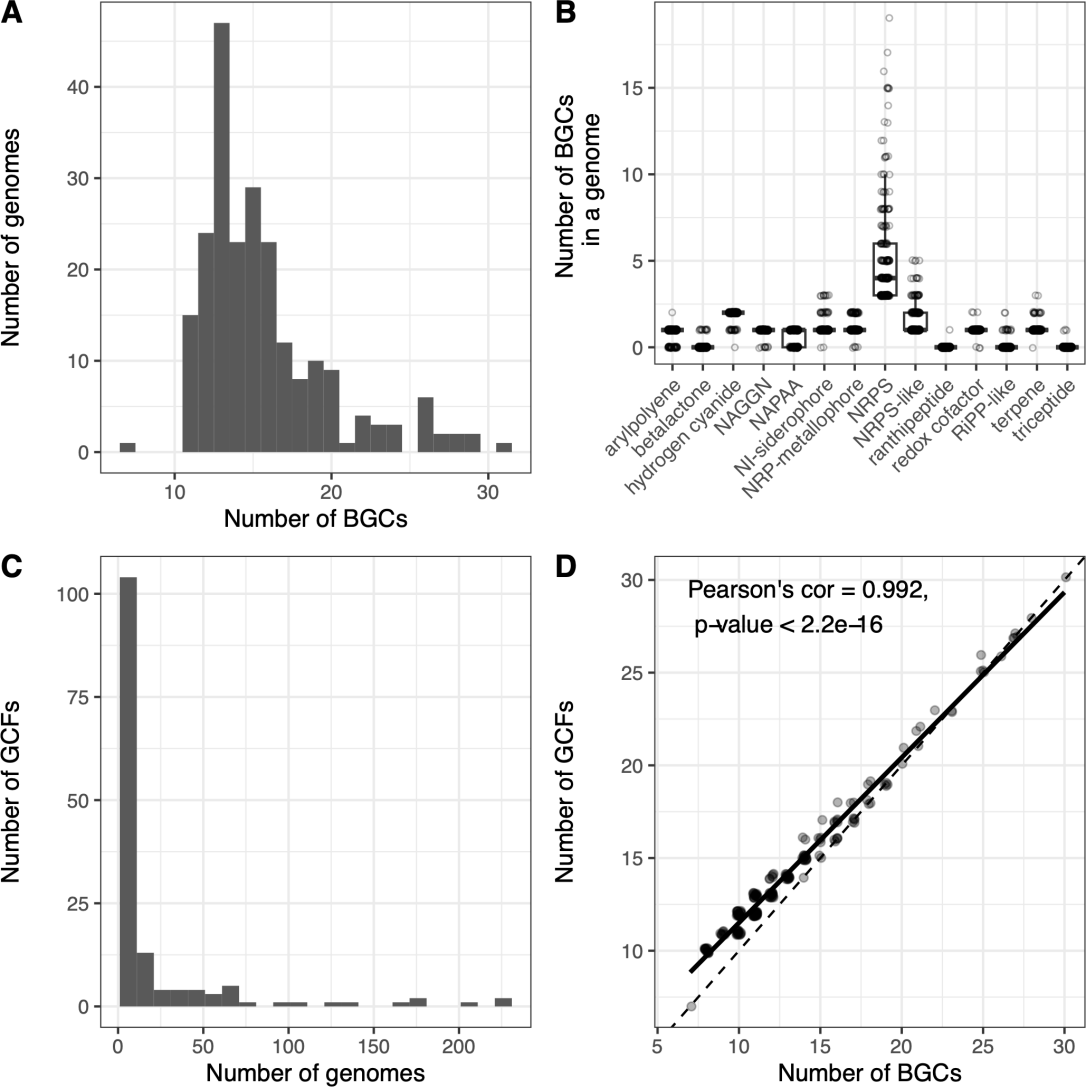
Specialized metabolite potential of *Pseudomonas viridiflava* associated with *Arabidopsis thaliana*. (**A**) Distribution of the number of BGCs predicted per genome. (**B**) Boxplot of the number of BGCs predicted per genome for each detected class. (**C**) Distribution of the number of genomes a given gene cluster family (GCF) is detected in. (**D**) Number of GCFs relative to all BCGs for each genome. Solid line: linear regression of the data; dashed line: unity. BGC: biosynthetic gene cluster (output from antiSMASH); GCF: gene cluster family (output from BiG-SCAPE).

We characterized the specialized metabolite potential of these 225 *A. thaliana*-associated *P. viridiflava* isolates with antiSMASH 7 (10), predicting their BGCs repertoire. When two or more BGC were predicted in the same genomic region, we counted each BGC independently. In total, 2,925 regions encoding 3,519 BGCs were predicted (Fig. 1). Genomes had on average 15.6 BGCs (median = 15, min = 7, max = 31; Fig. 2A). The percentage of BGCs on a contig’s edge ranged from 0% to 100%, with a mean of 41% and a median of 38% (Fig. S1A). We examined the relation between the total number of BGCs and the percentage of BGCs on a contig’s edge with the assembly statistics (Fig. S1). We found that genomes with more contigs and with lower completeness, i.e., of genomes of lower quality, had more predicted BGC in general, and more of the predicted BGCs were located on contig edges (Pearson’s correlation coefficient magnitude between 0.34 and 0.48).

Strains with disease data were interleaved with representative genomes and were not evenly distributed across the phylogeny (Fig. 1). Clusters of closely related genomes (based on 939 concatenated core genes) were observed, particularly within clades containing several strains included due to the availability of disease data and irrespective of their similarity to other genomes. However, genomes in these clusters differed in their BGC content, suggesting at least partial decoupling between core genome phylogeny and BGC repertoire. This is consistent with BGCs being common in the accessory genome, often mobile and important for ecological adaptation. Genomes harboring a high number of BGCs were distributed throughout the phylogenetic tree.

The predicted BGCs represented 14 of the 81 (17%) specialized metabolite chemical classes predicted by antiSMASH 7. The most abundant BGC classes were NRPS and NRPS-like, accounting for 43% of all the predicted BGCs (Table 1). These were the only BGC classes predicted in all isolates: every genome had at least three NRPS and one NRPS-like BGC (max = 19 and 5, respectively; Fig. 2B; Table 1). BGCs for hydrogen cyanides, NRP-metallophores, NI-siderophores, terpenes, redox cofactors, and NAGGNs were found in at least 90% of the genomes; together these classes represented an additional 46% of all the BGCs predicted in 225 ATUE5 genomes. On the other end of the frequency spectrum, BGCs for RiPP-like, betalactones, triceptides, and ranthipeptides were predicted in fewer than 10% of the genomes, representing only about 1% of all predicted BGCs.

**TABLE 1:** Number of predicted biosynthetic gene clusters per class in 225 plant-associated *P. viridiflava* genomes^*a*^

BGC class	*n*	% of all predicted BGCs	Min	Max	Mean	Median
NRPS	1,186	33.7	3	19	5.3	4
Hydrogen cyanide	426	12.1	0	2	1.9	2
NRPS-like	351	10.0	1	5	1.6	1
NRP metallophore	260	7.4	0	2	1.2	1
NI-siderophore	257	7.3	0	3	1.1	1
Terpene	243	6.9	0	3	1.1	1
Redox cofactor	225	6.4	0	2	1.0	1
NAGGN	216	6.1	0	1	1.0	1
Arylpolyene	177	5.0	0	2	0.8	1
NAPAA	142	4.0	0	1	0.6	1
RiPP-like	19	0.5	0	2	0.1	0
Betalactone	13	0.4	0	1	0.06	0
Triceptide	3	0.1	0	1	0.01	0
Ranthipeptide	1	0.03	0	1	0.00	0

^*a*^ BGC: biosynthetic gene cluster; NAGGN: N-acetylglutaminylglutamine amide; NAPAA: non-alpha poly-amino acids like e-polylysin; NI: NRPS-independent; NRPS: non-ribosomal peptide synthetases; RiPP: ribosomally synthesized and post-translationally modified peptide.

To determine the similarity among the predicted BGCs and to deduplicate them, we clustered all BGCs into gene cluster families (GCF) based on sequence similarity using BiG-SCAPE (39). A GCF contains BGCs that are predicted to produce chemically related specialized metabolites. The 3,519 BGCs were clustered into 148 GCFs (Table S2; Fig. S2). Almost one-third of the GCFs were singletons, i.e., they consisted of a single BGC found in only one genome. A GCF was present in a median of four genomes (mean = 21.6, IQR = 1–15, Fig. 2C). Nine GCFs were found in over 100 isolates, predicted to produce compounds of the classes arylpolyene, hydrogen cyanide, redox cofactor, NPRS, NRPS-like, NAPAA, and terpene. The two most prevalent families, which encoded a hydrogen cyanide and a redox cofactor, were almost fixed in the population, missing from only one and two genomes, respectively.

Only four GCFs had a reference specialized metabolite in the database of experimentally characterized BGCs, MIBiG (40): FAM_00334 (NRPS) had hits to biosurfactants cichofactin and syringafactin, FAM_00335 (NRPS) to virginiafactin, and FAM_00462 (NI-siderophore) to the siderophore achromobactin, all known from *Pseudomonas* species, while FAM_00458 (terpene) had hits to carotenoids from *Enterobacteriaceae* and *Pantoea* (Table S3). There was a strong and significant correlation between the number of BGCs and the number of GCFs predicted in a genome (Pearson’s correlation coefficient = 0.99, *P*-value < 0.0001; Fig. 2D). These results indicate that there is little redundancy in the BGCs encoded by a given *P. viridiflava* isolate, as BGCs of the same class usually belong to different GCFs. In addition, most of the specialized metabolites encoded by *P. viridiflava* genomes appear to be novel and/or have not yet been experimentally characterized. Their biological functions, therefore, remain unknown.

### Genes associated with biosynthetic gene clusters for specialized metabolites have a fitness cost for *P. viridiflava in planta*

Microbial specialized metabolites often mediate ecologically important functions, including the interaction with the hosts and with other microorganisms. Hence, it is not unlikely that the effect of carrying a BGC on bacterial fitness varies in different contexts. In this pathosystem, we have shown that bacterial load correlates positively with disease severity (27). To assess the fitness benefits or fitness costs of encoding a BGC *in planta*, we used a pool of BarSeq mutants in the *P. viridiflava* ATUE5:p25.C2 background (41), a highly virulent isolate on *A. thaliana* Ey15-2 (27, 41).

*Pseudomonas viridiflava* ATUE5:p25.C2 putatively encodes 11 BGCs in 8 regions of the genome, with region 6 encoding two completely-overlapping (i.e., chemical hybrid) BGCs and region 7 three partially-overlapping (i.e., neighboring hybrid) BGCs (Table 2; Fig. S3). There were 229 genes associated with these 11 BGCs, with the BarSeq mutant pool having strains with mutations in 222 of these genes (97%). The proteins encoded by the remaining 7 BGC-associated genes were annotated by antiSMASH 7 as hypothetical protein (region 4), transcriptional regulator LiaR, protein MbtH and glutamate tRNA ligase (region 6), and NAD-dependent glycerol dehydrogenase, transcriptional regulator LsrR and hypothetical protein (region 7).

**TABLE 2:** Biosynthetic gene clusters (BGCs) predicted in *P. viridiflava* ATUE5:p25.C2^*a*^

Region	Type	No. of genes in BGC	No. of genes represented in mutant pool	DAMs Ey15-2	DAMs Col-0
Region 1	NRPS	32	32	0	6
Region 2	NAGGN	12	12	1	2
Region 3	NRPS-like	30	30	0	4
Region 4	Hydrogen-cyanide	10	9	0	0
Region 5	Redox-cofactor	17	17	3	1
Region 6	NRP-metallophore, NRPS	57	54	10	6
Region 7	Terpene, NRPS, NI-siderophore	60	57	9	5
Region 8	Hydrogen-cyanide	11	11	0	1
Total genes		229	222	23	25

^*a*^ BGC: biosynthetic gene cluster; DAM: differentially-abundant BarSeq mutant; NAGGN: N-acetylglutaminylglutamine amide; NI: NRPS-independent; NRPS: non-ribosomal peptide synthetases. Underlined numbers indicate that the number of DAMs was greater than expected by chance (empirical *P*-value ≤ 0.05).

**Fig 3:**
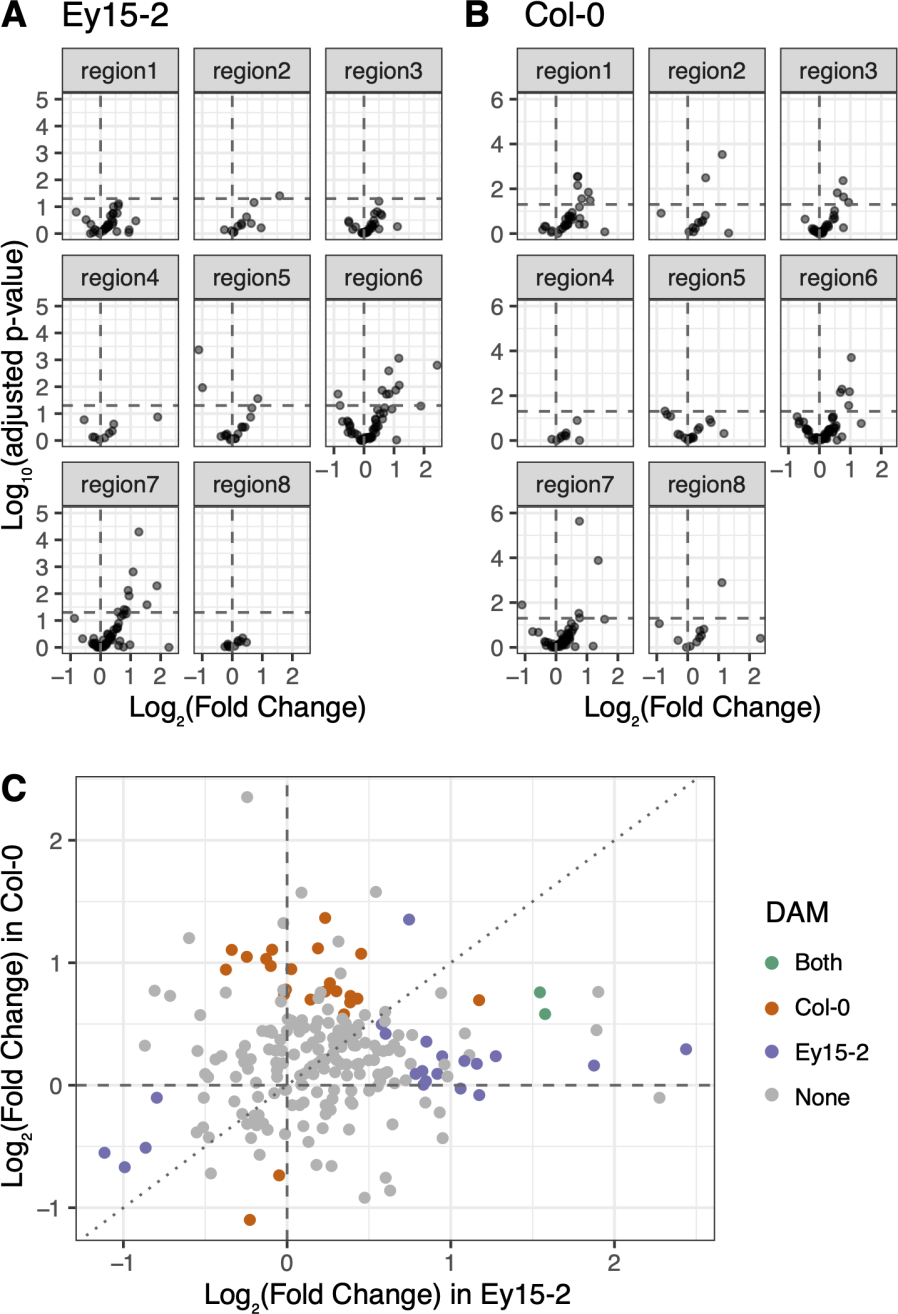
Behavior of BGC mutants in planta. (**A and B**) Volcano plots of abundance change (mutant abundance *in planta* compared to inoculum) of mutants defective in 222 BGC-associated genes, 3 days after infection of Ey15-2 (**A**) or Col-0 (**B**). Dashed lines indicate no change in abundance (*x*-axis, log_2_ fold change = 0) and the significance threshold (*y*-axis, *P* = 0.05). (**C**) Correlation between abundance changes in Ey15-2 and Col-0. Colors indicate whether changes were significant in one or the other accession or in both The dotted line indicates perfect correlation. DAM: differentially abundant mutant.

We used this p25.C2 mutant pool to infect axenic *A. thaliana* Ey15-2 and Col-0 plants. RB-TnSeq (42) was applied to the pooled mutants used as inoculum and to infected plants 3 days after infection. We estimated the fitness of BGC-associated genes from the ratio of barcode reads in the material harvested from plants compared to the initial inoculum. We identified BGC-associated genes whose mutants changed in abundance in the BarSeq pool upon infection of *A. thaliana* plants with DESeq2 (43). The estimated fold change of most BGC-associated BarSeq mutants was positive across the two *A. thaliana* accessions Col-0 and Ey15-2 (Fig. 3A and B; Tables S4 and S5). We identified 25 and 23 differentially-abundant mutants (DAMs) for the 222 BGC-associated genes in Col-0 and Ey15-2 infections, respectively, defined as having a log_2_ fold change with an adjusted *P*-value ≤ 0.05 (Table 2; Fig. S3). To validate whether the identified DAMs represented a biological signal or a random occurrence due to the large number of genes in a BGC, we calculated the empirical *P*-value for each BGC region from 1,000 random sets of contiguous genes corresponding to the size of each region. The observed number of DAMs in region 6 for Ey15-2 was greater than expected by random chance (empirical *P*-value ≤ 0.05).

The longest BGCs, with the largest number of both associated genes and DAMs, were the 102 kb-long region 6 encoding an NRP-metallophore and an NRPS hybrid, and the 97 kb-long region 7 encoding a terpene, an NRPS, and an NI-siderophore hybrid. We identified six DAMs in region 1 and four DAMs in region 3 after Col-0 infection, but none after Ey15-2 infection (Fig. 3; Fig. S1). Only two DAMs were shared between Ey15-2 and Col-0, one in region 2 and one in region 7.

We compared the fitness of each mutant in Col-0 infections against Ey15-2 infections and found no significant correlation between the two infection environments (Pearson’s correlation coefficient = 0.07, *P*-value of 0.31) (Fig. 3C). Moreover, the direction of the fold change was not always consistent between the two accessions, with some mutants with increased abundance in Ey15-2 having a negative or no change in Col-0 and vice versa. Taken together, these results suggest that BGC fitness effects are strongly dependent on the host environment.

We found that the abundance of most BGC mutants increased *in planta*: 74% and 68% of the BGC mutants increased in Col-0 and Ey15-2, respectively, indicating improved fitness upon BGC disruption. The abundance of only two and four mutants was reduced in Col-0 and Ey15-2, respectively. We compared the distribution of fold changes of strains with mutations in core BGC biosynthetic genes and non-core BGC biosynthetic genes (i.e., additional biosynthetic, transport-related, regulatory and other genes) as well as in non-BGC-associated genes. The fold change of core biosynthetic genes tended to be higher than that of non-core biosynthetic genes and non-BGC-associated genes although the difference was not significant (Kolmogorov-Smirnov, *P*-value > 0.05, Fig. S4). Overall, contrary to our initial expectations, the increased ability of the BarSeq mutants to grow *in planta* suggests that BGC production can reduce bacterial fitness.

BGC-associated genes could impose a fitness cost on *P. viridiflava* in general, not only during plant infection. To assess this possibility, we grew the p25.C2 mutant pool on rich media for 24 h and determined the fitness of BGC-associated genes’ mutants. We recovered mutants in 213/229 (93%) BGC-associated genes; 15 of these genes had low counts and were excluded from further analyses. The distribution of fold changes of BGC-associated genes was not significantly different from the fold changes of non-BGC-associated genes (two-sided Kolmogorov-Smirnov test, *P*-value = 0.36). There were 47/4156 DAMs, two of which corresponded to BGC-associated genes, one from region 5 and one from region 7 (Fig. S5). The DAM on region 5 was also identified in *A. thaliana* Col-0 infection. Taken together, these results suggest that the fitness cost associated to BGC-associated genes is specific to *in planta* colonization and growth.

### The presence/absence of biosynthetic gene clusters correlates with disease severity on *A. thaliana*

We had hypothesized that specialized metabolites contribute to *P. viridiflava* ATUE5 pathogenicity in *A. thaliana*. However, our RB-Tnseq experiments suggested instead that BGCs, particularly core biosynthetic genes, are more likely to carry a fitness cost rather than a fitness benefit for *P. viridiflava in planta*. Tn-seq experiments and their variants, including RB-TnSeq, measure fitness in a population-dependent context and can underestimate the fitness advantage of compounds that are public goods, i.e., products that are secreted and affect not only the producer bacterium but also everyone else in the environment. Many specialized metabolites are secreted and thus are considered public goods for members of a bacterial population that co-exist in a shared environment (44).

**Fig 4:**
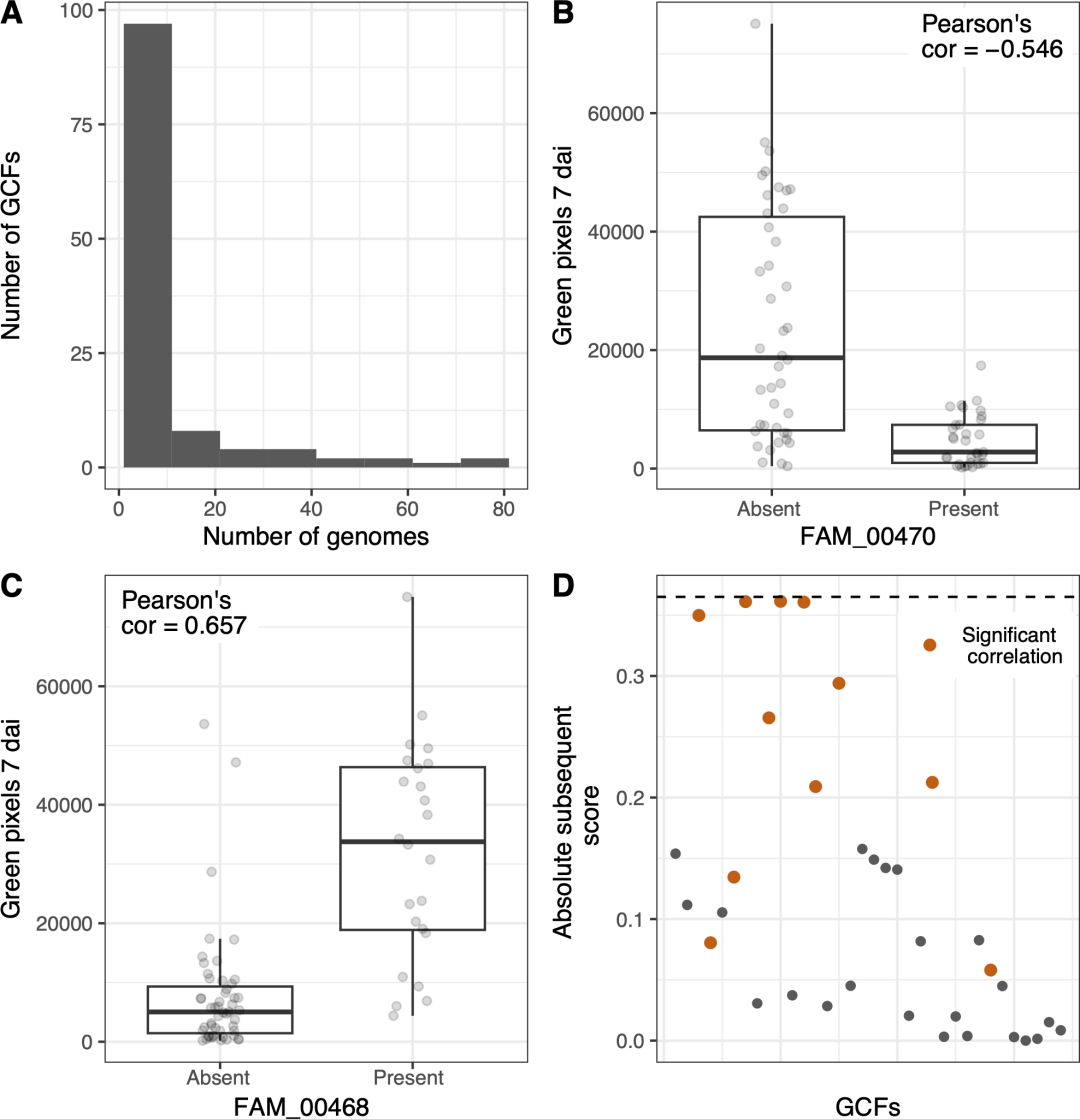
Association of gene cluster families presence/absence with disease severity *in planta****.*** (**A**) Distribution of the number of genomes a given GCF is detected in a subset of 75 *P. viridiflava* genomes. (**B**) Boxplot of the number of green pixels recorded 7 days after infection with *P. viridiflava* isolates where FAM_0470 was either absent or present. (**C**) Boxplot of the plant’s green pixels 7 days after infection with *P. viridiflava* isolates where FAM_0468 was either absent or present. (**D**) Manhattan plot of treeWAS subsequent test for 34 GCFs found in at least four but fewer than 67 *P. viridiflava* isolates. Orange points indicate GCFs whose presence/absence was significantly correlated with plant size as inferred from the number of green pixels (Table 3). GCF: Gene cluster family; dai: days after infection.

Because pooled mutant infections allow for cheaters to exploit secreted metabolites produced by other strains, we next tested whether BGC effects persisted when plants were infected with individual isolates. To this end, we reanalyzed an available experimental data set where axenic *A. thaliana* Ey15-2 plants were infected with 75 *P. viridiflava* ATUE5 isolates in single-isolate infections, and plant size was measured after 7 days after infection as a proxy for disease severity (27). In this context, only the specialized metabolites encoded by the infecting isolate are available, and thus, there are no public goods from genetically different strains. The distribution of the 120 GCFs in these 75 isolates was similar to that of the entire collection, with a median of 12 GCFs per genome (mean = 13.7, min = 7, max = 30; Fig. 4A). One-third of the GCFs (35%) were restricted to a single isolate. We determined the correlation between the presence of a GCF and plant size after infection, including only those GCFs present in at least 5%, but no more than 90% of the isolates. This resulted in 34 testable GCFs, including seven GCFs present in *P. viridiflava* ATUE5:p25.C2. For 11 GCFs, their presence correlated with plant size after infection (Pearson’s correlation coefficient between −0.546 and 0.769, adjusted *P*-value ≤ 0.05, Table 3; Fig. 4B and C). Only one of these families (FAM_00334) had hits in the MIBiG database, to biosurfactants syringafactin and cichofactin. For two GCFs, FAM_00392 and FAM_00470, the Pearson’s correlation coefficient was negative, suggesting that the presence of these specialized metabolite gene clusters is associated with increased disease severity (measured as smaller plant size after infection) on *A. thaliana* Ey15-2. These GCFs correspond to regions 1 and 2 in *P. viridiflava* ATUE5:p25.C2, respectively. An additional GCF present in *P. viridiflava* ATUE5:p25.C2 had a significant correlation with disease severity, in this case positive: FAM_00355, corresponding to region 6. Region 6 had more BGC-associated genes that changed abundances in Ey15-2 than expected by chance (Table 2). Three GCFs from ATUE5:p25.C2 were present in >90% of the isolates and were not included in the correlation analysis (FAM_00472—region 4, FAM_00476—region 5 and FAM_00471 – region 8). The remaining four GCF present in ATUE5:p25.C2 had no significant correlation with disease severity (FAM_00387—region 3 and FAM_00335, FAM_00462 and FAM_00458—region 7).

**TABLE 3:** Gene cluster families correlated with plant size after infection^*a*^

GCF	Product class	Number of isolates (%)	Pearson’s correlation coefficient	Adjusted *P*-value
FAM_00470^*c*^	NAGGN	33 (44)	−0.546	3.51 x 10^−6^
FAM_00392^*c*^	NRPS	18 (24)	−0.406	1.45 x 10^−3^
FAM_00344	NRPS	7 (9)	0.277	4.96 x 10^−2^
FAM_00355^*c*^	NRPS	42 (56)	0.278	4.96 x 10^−2^
FAM_00463	NI-siderophore	33 (44)	0.312	2.422 x 10^−2^
FAM_00334^*b*^	NRPS	20 (27)	0.326	1.81 x 10^−2^
FAM_00384	NAPAA	28 (37)	0.420	1.01 x 10^−3^
FAM_00455	Arylpolyene	49 (65)	0.505	2.56 x 10^−5^
FAM_00473	Betalactone	11 (15)	0.632	1.31 x 10^−8^
FAM_00468	NAGGN	24 (32)	0.657	2.63 x 10^−9^
FAM_00390	NRPS	22 (29)	0.769	2.71 x 10^−14^

^*a*^ GCF: gene cluster family; BGC: biosynthetic gene cluster.

^*b*^ Hits to syringafactin and cichofactin in the MIBiG database.

^*c*^ Families encoded by isolate p25.C2.

Population structure could influence the results of our correlation analysis, reflecting lineage rather than causality. Hence, we then applied treeWAS, a GWAS approach that corrects for population structure (45) on the same data set of 34 GCFs in 75 ATUE5 isolates. We did not find any GCF significantly associated with disease severity with treeWAS (Fig. 4D) although the GCFs with the highest absolute subsequent score were also significantly correlated with plant size. We, thus, cannot decisively conclude that these BGCs are virulence factors *in planta,* but they are priority candidates for functional validation.

## DISCUSSION

The genus *Pseudomonas* is known for its ability to produce many specialized metabolites (28, 29), which are often hypothesized to play a role in the interaction with other organisms and the environment. Here, we show that this also applies to *Arabidopsis thaliana*-associated isolates of *P. viridiflava*, which encode a remarkably diverse and largely uncharacterized repertoire of BGCs, dominated by NRPS and NRPS-like families. Despite this extensive biosynthetic potential, RB-TnSeq experiments revealed that disrupting BGC-associated genes generally improved bacterial growth *in planta*, indicating that specialized metabolite production often imposes a fitness cost during infection. This cost varied strongly with host genotype, suggesting substantial genotype-by-genotype interactions. Complementary analyses correlating BGC presence–absence with disease severity identified a subset of BGC families associated with altered disease severity, suggesting several candidates whose products may limit pathogenicity but be beneficial in other ecological contexts.

In our collection of 225 genomes, antiSMASH 7.1.0 (10) predicted 3,519 BGCs from 14 different chemical classes that clustered into 148 GCFs based on sequence similarity networks (39). Fragmented genomes could lead to the overestimation of BGCs due to a single BGC being distributed over multiple contigs. It is likely that the large specialized metabolite potential of *P. viridiflava* is associated with the diverse ecological niches it occupies as a generalist and opportunist pathogen, which include diverse hosts and non-plant environments (46, 47). That almost 30% of the GCFs are present in only a single isolate and that only nine GCFs are found in over 100 isolates support this hypothesis.

Fewer than 5% of the 148 gene cluster families had a closely related hit in the MIBiG database of experimentally characterized specialized metabolites. It is important to note, however, that even though the MIBiG database is the largest database of experimentally validated BGCs to date, currently, it contains only 3,013 hand-curated BGCs and their products, 75% of which are either PKS or NRPS (40, 48). Although the use of different software and database versions makes comparisons difficult across previous studies, finding MIBiG matches for only a few BGCs encoded by the bacteria of interest seems to be the norm rather than the exception (12, 16, 49, 50). Indeed, in an analysis of over 200,000 bacterial genomes and metagenome-assembled genomes (MAGs), 97% of the predicted specialized metabolites had not yet been experimentally characterized (31).

PKS and PKS/NRPS hybrids, such as the antimicrobial mupirocin and the toxin syringolin, are known to be encoded by *Pseudomonas* isolates, including plant-associated isolates (12, 29, 30, 32, 36). However, no PKS or PKS/NRPS hybrid was predicted in the *P. viridiflava* isolates we analyzed. Half of the predicted BGCs (51%) were related to NRPS (classes NRPS, NRPS-like, and NRP metallophore), in agreement with previous genome mining studies of the *Pseudomonas* genus (12, 30, 34). All isolates encoded at least three NRPS and one NRPS-like BGC, making these BGC classes the only ubiquitous ones in the genomes we analyzed.

NRPS can function as phytotoxins, antimicrobials, siderophores, and surfactants, among others (51). Clustering all predicted NRPS by sequence similarity (39) resulted in 121 GCF, but only two of these families had hits to the MIBiG database (40). These hits suggest that *P. viridiflava* encodes metabolites similar to the biosurfactants syringafactin and cichofactin and to the yet-uncharacterized virginiafactin. We expect our isolates to produce cichofactin and not syringafactin, as a recent characterization of the lipopeptide diversity of the *P. syringae* species complex found that isolates belonging to phylogroups 7 and 8, which include *P. viridiflava* (52), produce cichofactin and not syringafactin (33). Although Bricout and colleagues (33) reported that isolates are either monoproducers of factins or multiproducer of the three lipopeptides families (factins, mycins, and peptides), at least eight of our isolates encode BGCs closely related to both cichofactin/syringafactin and virginiafactin. The simultaneous production of both factins by a single isolate still needs to be demonstrated. There were no hits to toxins included in the MIBiG database, confirming our previous finding that *P. viridiflava* does not encode coronatine, mangotoxin, phaseolotoxin, syringomycin, syringopeptin nor tabtoxin (24).

With antiSMASH and its predecessors, it has become increasingly straightforward to predict BGCs in microbial genomes. Tn-seq provides a complementary high-throughput approach to test the fitness of BGC-associated genes at scale, although most of these studies have been done *in vitro*, and only a few in eukaryotic hosts (35). Between 2009, when Tn-Seq was first described, and mid-2023, fewer than 20 Tn-Seq studies with plant hosts were published (35), but 100 studies with animal hosts.

In two genetically distinct *A. thaliana* accessions, Col-0 and Ey15-2, we found that BGCs mostly reduced rather than increased *P. viridiflava* fitness. This was not the case in rich media, where we identified only two DAMs (1% of BGC-associated genes vs 10% *in planta*), suggesting this is a plant-specific finding. Surprisingly, the fitness costs of BGCs were strongly host dependent, and only two BGC mutants were differentially abundant in both Ey15-2 and Col-0, while the remaining 44 BGC mutants differed significantly in their abundance in only one of the two hosts. Thus, the well-documented genotype-by-genotype interactions in the *A. thaliana-P. viridiflava* pathosystem (27) extend also to BGCs. Despite it being generally stated that BGCs and their products provide a fitness advantage, this is not always the case. In line with our results showing a fitness cost of BGCs for growth *in planta*, almost 90% of BGC mutants in the plant pathogenic fungi *Fusarium graminearum* retained wild-type virulence on wheat (53). Mutation of some BGC resulted in bigger lesions in wheat coleoptiles, but this was not statistically significant. In addition, it has been reported that toxin syringomycin is required for competitive fitness of *P. syringae* pv. *syringae* B728a in the apoplast of common bean, while mutants in the syringopeptin and syringolin toxins do not have significant apoplastic fitness defects (54). Mutants in phytotoxin synthesis and transport genes even have higher fitness than the wild-type strain in at least one of three plant hosts (common bean, lima bean, and pepper) in a host-specific manner (55).

We were surprised by the cost associated with BGC-associated genes, a result that contradicted our starting hypothesis. Nonetheless, an analysis of key BGCs in *P. fluorescens* found that only 2 out of 26 Tn-seq mutants had a growth disadvantage in at least 1 of over 100 conditions (47, 56). As specialized metabolites can mediate not only host-microbe interactions but also microbe-microbe and microbe-environment interactions, one potential explanation is that many of the BGCs have functions unrelated to host colonization and/or contribute to successful plant colonization by acting outside of the host, i.e., on the surrounding microbial communities. An example of this is the shift in the composition of a protective synthetic community induced by an opportunistic *Pseudomonas* root pathogen, which requires the production of two specialized metabolites, a siderophore and an antibiotic (57). This remodeling of the synthetic community contributes to an increase in root colonization by the opportunistic pathogen (57). Another possible reason for the observed fitness cost of BGC-associated genes is that specialized metabolites can be detected by the host and/or by host-associated microbes, triggering a defense response, and/or be toxins that kill the host and, thus, lead to a reduction in the bacterial counts. The *Pseudomonas* siderophore pyochelin, for example, causes a root-associated *Bacillus* isolate to increase its production of antibacterials (58).

A caveat to Tn-seq experiments is that they can lead to underestimates of fitness costs from genes that encode secreted public goods (35, 59, 60), such as siderophores, biosurfactants, and toxins (61). Public goods are exploited by cheaters, individuals of the population that benefit from a public good without contributing to the production of said good, i.e., at no (fitness) cost (61). It has been shown, for instance, that mutants with defects in siderophore production have superior fitness than wild-type isolates when co-cultured, where they can cheat, but lower fitness in mono-cultures (62, 63). In addition, specialized metabolites are energetically expensive and divert resources from primary metabolism, so they can be detrimental for the producer (64) under certain conditions, similar to antibiotic resistance genes having a cost in the absence of the antibiotic (65, 66).

To overcome a possible underestimation of a BGC fitness in our RB-TnSeq experiments, we followed an orthogonal approach by examining the outcome of infections with genetically diverse *P. viridiflava* isolates that naturally vary for BGC presence. Eleven of the 34 tested GCFs correlated with disease severity in the *A. thaliana* accession Ey15-2. Similar to the results of the RB-TnSeq experiments, most GCFs were correlated with reduced disease severity, i.e., larger plant size measured in green pixels, but two were associated with increased disease severity. Of the 11 GCFs encoded by *P. viridiflava* ATUE5:p25.C2, 7 were included in this analysis; the remaining 3 were highly prevalent and could not be tested. Our results suggest that the BGCs corresponding to regions 3, 6, and 7 are not virulence factors during *P. viridiflava* infection of *A. thaliana*; while regions 1 and/or 2 could play a role in the infection and further experiments are required to confirm a role in virulence. This is also the case for regions 4, 5, and 8, which were highly prevalent in our set of *P. viridiflava* isolates and, thus, could not be tested with the statistical methods we used. Region 5 is of particular interest, since we found DAMs with negative fold changes in this region in both host genotypes.

We found four NRPS associated with reduced disease severity in infected *A. thaliana*, including the one predicted to be related to the biosurfactant cichofactin. This product, therefore, does not appear to be a virulence factor in *A. thaliana* (at least under the conditions of mono-association in an axenic system) despite previous reports (6). Meanwhile, the two GCFs negatively correlated with disease severity are potential virulence factors in *A. thaliana* infection and should be experimentally validated. One encodes a NAGGN (N-Acetylglutaminyl glutamine amide), a modified dipeptide with an important role in bacterial osmoregulation (67), and the other encodes a NRPS.

While our uncorrected correlations identified BGC associated both with increased and decreased disease severity, no significant association remained after phylogenetic correction. This could reflect limited power due to the number of isolates and/or non-random sampling. Hence, these candidate BGCs and the secondary metabolites they produce should be tested experimentally to confirm whether they play a role during *in planta* infection, particularly the two GCFs associated with increased disease severity. Although simple, a similar correlation approach identified BGCs potentially associated with *Pseudomonas* inhibition of the potato pathogen *Streptomyces scabies* (36); and further experimental characterization confirmed a role of two BGCs, a cyclic lipopeptide and of hydrogen cyanide, on the *Pseudomonas-Streptomyces* interaction (36).

Our study is not without limitations. antiSMASH tends to overpredict BGC-associated genes (68), resulting in the inclusion of adjacent genes in a predicted BGC that might not be involved in the production of its specialized metabolite, and/or that disruption of some BGC-associated does not prevent the production of the specialized metabolite (69). Experimental and functional characterization of BGCs, while technically challenging, is necessary to validate our results. We tested the fitness effect of BGC-associated genes in rich media and *A. thaliana*, but we cannot discard that BGCs confer a fitness advantage in other environments, including in microbe-microbe interactions. Indeed, Wang et al. recently demonstrated that the kosinostatin BGC confers a fitness advantage to *Streptomyces albidoflavus* in liquid culture but not in soil, and in the absence of a sensitive strain, carrying the BGC incurred in a fitness cost even in liquid media (70).

In summary, our results challenge the common assumption that specialized metabolite production enhances pathogen success in plant hosts. Instead, while many *P. viridiflava* isolates encode well-known specialized metabolites that serve as virulence factors, such as hydrogen cyanide, the biosurfactant cichofactin, and the siderophore achromobactin, we find that specialized metabolites seem to have a limited role in the virulence of *P. viridiflava* on *A. thaliana*, at least when tested in mono associations. As *P. viridiflava* is a generalist that is also found in water-related environments including rain drops, snowpack, rivers, and lakes (46), there is ample opportunity for its specialized metabolites to provide fitness benefits in a non-plant context. Future experiments should include the characterization of the specialized metabolites produced by *P. viridiflava* isolates and determine their production upon plant infection, the validation of the fitness cost of BGCs in single-isolate *A. thaliana* infections using, e.g., knock-outs as well as in co-infections, and the interaction between BGCs and host genotypes.

## MATERIALS AND METHODS

### Selection of representative *Pseudomonas* genomes

The genomes in this study were described before (24). We selected representative genomes based on gene content similarity, i.e., presence/absence pattern of gene orthology groups. For this, we first assessed the completeness of each genome using BUSCO version 3.0.2 (71). Of 1,514 genomes, 1,338 had a single-copy completeness score equal to or above 95%. These genomes were clustered using the GeneContRep algorithm implemented in the PanGene-O-Meter package (3,8). Briefly, the clustering procedure is as follows.

1. Sort all genome assemblies by their total length, from the longest to the shortest.
2. In each iteration, set the longest assembly as the “representative” of a newly formed cluster.
3. Assign members to the newly formed cluster based on the similarity of their orthology groups presence-absence profiles. Iterate over all genomes not yet assigned to a cluster and compute the Jaccard similarity coefficient (72) between the presence-absence profile of orthology groups (obtained from PanX assignments [24]) of said genome and the cluster “representative.” The Jaccard similarity coefficient was calculated as:
$$J\;=\frac{M_{11}}{M_{01}+M_{10}+M_{11}},$$
  where *M*_11_ is the number of orthology groups shared between the strains; *M*_10_ and *M*_01_ are orthology groups present in the representative and the assessed genome, respectively.
  If the Jaccard similarity coefficient is greater or equal to 0.99, assign the genome to the cluster and remove it from the list.
4. Repeat steps 2 and 3 until all genomes are assigned to a cluster.

### Annotation of biosynthetic gene clusters

We used antiSMASH 7.1.0 (10) to predict biosynthetic gene clusters (BGCs) on each genome. MultiSMASH 0.3.1 was used to tabulate antiSMASH results (73). When more than one BGC was predicted in a region, each BGC was counted independently in its corresponding class. For example, a region with “NRPS, terpene, siderophore” accounted for three BGCs in our analyses. Sequence similarity networks of the BGC were generated using BiG-SCAPE (39) version 2.0.0b6, available at github.com/medema-group/BiG-SCAPE with parameters --hybrids-off and --include-singletons. We included entries from the Minimum Information about a Biosynthetic Gene (MIBiG) database (40) with parameter --mibig-version 4.0, providing manually-curated BGC annotations with known functions to the gene cluster families identified in the *P. viridiflava* genomes. The number and distribution of BGCs were plotted on the core genome maximum-likelihood tree (24) using iToL (74).

### Plant infections with a pool of RB-TnSeq *P. viridiflava* ATUE5:p25.C2 mutants

BarSeq mutants were generated in *P. viridiflava* ATUE5:p25.C2 (40). Briefly, we conjugated *P. viridiflava* ATUE5:p25.C2 with the *E. coli* conjugation donor WM3064 harboring the pHLL250 mariner transposon vector library (AMD290) (68). Stocks of the mutant pool were stored at −80°C in 10% glycerol at an OD_600_ of 1.0 and were used to infect *A. thaliana*.

Col-0 and Ey15-2 wild-type *A. thaliana* plants were grown under long-day conditions (16 h light, 8 h dark) in an AR41L3 Percival growth chamber with 60% intensity of SciWhite LED lights. Seedlings were grown in 24-well plates on ½ Murashige & Skoog medium with vitamins and MES buffer. Thirteen-day-old seedlings were used for the infections. The bacterial suspension of pooled *P. viridiflava* ATUE5:p25.C2 mutants was diluted from an OD_600_ of 1.0 to an OD_600_ of 0.02 using 10 mM MgSO_4_. Each plant was infected with 200 μL of this bacterial suspension. As a negative control, plants were infected with 10 mM MgSO_4_ alone. Samples were collected in 2 mL deep-well plates 3 days after infection, frozen and then ground using two 5 mm glass beads. DNA was extracted using Qiagen buffers. Each biological sample contained two infected plants.

Random barcode transposon-site sequencing (RB-TnSeq) (42) was performed to determine the fitness of each mutant in the pool. Barcode sequence data from the stock mutant pool and the infected plants were obtained by multiplexing on a partial NovaSeq 6000 lane at Novogene Corporation Inc. Fitness was calculated from the abundance of reads in the plant material vs the initial inoculum pool and analyzed with the DESeq2 package (43) in R v4.4.0 (75) to identify differentially abundant mutants (DAMs).

To determine the statistical significance of the number of mutants for a BGC region (DAMs), we calculated the empirical *P*-value (76). For this, we generated 1,000 random sets of contiguous genes of comparable size to each BGC region and determined how many of the mutants in each random set were differentially abundant (adjusted *P*-value ≤ 0.05). We then calculated the probability of finding as many or more DAMs in the 1,000 random sets for each region compared to the observed, i.e., experimental, number of DAMs in the same region. We used this probability as the empirical *P*-value.

### Growth of a pool of RB-TnSeq *P. viridiflava* ATUE5:p25.C2 mutants *in vitro*

The bacterial suspension of pooled RB-TnSeq mutants was let to recover for 2 h at 28°C before being diluted to an OD_600_ of 0.02 and grown at 28°C in Luria-Bertani (LB) medium with 100 μg/mL of nitrofurantoin and 50 μg/mL of kanamycin for 24 h. RB-Tnseq and fitness calculations were done as described above to identify DAMs.

### Correlation/association of green pixels with gene cluster families

We had previously generated data on virulence measured as green pixels of 75 genetically diverse *P. viridiflava* ATUE5 isolates on *A. thaliana* Ey15-2 (27). We calculated Pearson’s correlation coefficients between the presence/absence matrix of GCFs encoded by 5% to 90% isolates and the mean green pixels 7 days after infection as a proxy for fitness *in planta*. The *P*-value was adjusted for multiple comparisons using the Benjamin-Hochberg method in R v4.4.0 (75).

Genome-wide associations between the presence/absence matrix of GCFs and green pixels after infection were obtained using treeWAS (45) in R v4.4.0 (75), with 100 permutations. When a GCF had more than one copy per genome, the corresponding cell in the matrix was changed to 1 to comply with treeWAS requirements. The three association tests included in treeWAS, terminal, simultaneous and subsequent, were run. A GCF was considered significantly associated with fitness *in planta* when the adjusted *P*-value ≤ 0.05.

## Supplementary Material

Reviewer comments

## Data Availability

Genome IDs are given in Table S1. Green pixel data are from reference 27. Other materials and data that are reasonably requested will be made available in a timely fashion.

## SUPPLEMENTAL MATERIAL

Supplemental figures msystems.00212-26-s0001.pdf Figures S1 to S5.

Supplemental tables msystems.00212-26-s0002.xlsx Tables S1 to S5.

OPEN PEER REVIEW reviewer-comments.pdf An accounting of the reviewer comments and feedback.

ASM does not own the copyrights to Supplemental Material that may be linked to, or accessed through, an article. The authors have granted ASM a non-exclusive, world-wide license to publish the Supplemental Material files. Please contact the corresponding author directly for reuse.

## References

[1] O’Brien J, Wright GD. 2011. An ecological perspective of microbial secondary metabolism. Curr Opin Biotechnol 22:552–558. doi:10.1016/j.copbio.2011.03.010 21498065

[2] Etalo DW, Jeon J-S, Raaijmakers JM. 2018. Modulation of plant chemistry by beneficial root microbiota. Nat Prod Rep 35:398–409. doi:10.1039/c7np00057j 29722774

[3] Raaijmakers JM, De Bruijn I, Nybroe O, Ongena M. 2010. Natural functions of lipopeptides from Bacillus and Pseudomonas: more than surfactants and antibiotics. FEMS Microbiol Rev 34:1037–1062. doi:10.1111/j.1574-6976.2010.00221.x 20412310

[4] Taguchi F, Suzuki T, Inagaki Y, Toyoda K, Shiraishi T, Ichinose Y. 2010. The siderophore pyoverdine of Pseudomonas syringae pv. tabaci 6605 is an intrinsic virulence factor in host tobacco infection. J Bacteriol 192:117–126. doi:10.1128/JB.00689-09 19854904 PMC2798240

[5] Berti AD, Greve NJ, Christensen QH, Thomas MG. 2007. Identification of a biosynthetic gene cluster and the six associated lipopeptides involved in swarming motility of Pseudomonas syringae pv. tomato DC3000. J Bacteriol 189:6312–6323. doi:10.1128/JB.00725-07 17601782 PMC1951903

[6] Pauwelyn E, Huang C-J, Ongena M, Leclère V, Jacques P, Bleyaert P, Budzikiewicz H, Schäfer M, Höfte M. 2013. New linear lipopeptides produced by Pseudomonas cichorii SF1-54 are involved in virulence, swarming motility, and biofilm formation. Mol Plant Microbe Interact 26:585–598. doi:10.1094/MPMI-11-12-0258-R 23405865

[7] Medema MH, Kottmann R, Yilmaz P, Cummings M, Biggins JB, Blin K, de Bruijn I, Chooi YH, Claesen J, Coates RC, et al.. 2015. Minimum information about a biosynthetic gene cluster. Nat Chem Biol 11:625–631. doi:10.1038/nchembio.1890 26284661 PMC5714517

[8] Dinglasan JLN, Otani H, Doering DT, Udwary D, Mouncey NJ. 2025. Microbial secondary metabolites: advancements to accelerate discovery towards application. Nat Rev Microbiol 23:338–354. doi:10.1038/s41579-024-01141-y 39824928

[9] Medema MH, Blin K, Cimermancic P, de Jager V, Zakrzewski P, Fischbach MA, Weber T, Takano E, Breitling R. 2011. antiSMASH: rapid identification, annotation and analysis of secondary metabolite biosynthesis gene clusters in bacterial and fungal genome sequences. Nucleic Acids Res 39:W339–46. doi:10.1093/nar/gkr466 21672958 PMC3125804

[10] Blin K, Shaw S, Augustijn HE, Reitz ZL, Biermann F, Alanjary M, Fetter A, Terlouw BR, Metcalf WW, Helfrich EJN, van Wezel GP, Medema MH, Weber T. 2023. antiSMASH 7.0: new and improved predictions for detection, regulation, chemical structures and visualisation. Nucleic Acids Res 51:W46–W50. doi:10.1093/nar/gkad344 37140036 PMC10320115

[11] Steinke K, Mohite OS, Weber T, Kovács ÁT. 2021. Phylogenetic distribution of secondary metabolites in the Bacillus subtilis species complex. mSystems 6:e00057-21. doi:10.1128/mSystems.00057-21 33688015 PMC8546965

[12] Saati-Santamaría Z, Selem-Mojica N, Peral-Aranega E, Rivas R, García-Fraile P. 2022. Unveiling the genomic potential of Pseudomonas type strains for discovering new natural products. Microb Genom 8:000758. doi:10.1099/mgen.0.000758 35195510 PMC8942027

[13] Donia MS, Cimermancic P, Schulze CJ, Wieland Brown LC, Martin J, Mitreva M, Clardy J, Linington RG, Fischbach MA. 2014. A systematic analysis of biosynthetic gene clusters in the human microbiome reveals a common family of antibiotics. Cell 158:1402–1414. doi:10.1016/j.cell.2014.08.032 25215495 PMC4164201

[14] Paoli L, Ruscheweyh H-J, Forneris CC, Hubrich F, Kautsar S, Bhushan A, Lotti A, Clayssen Q, Salazar G, Milanese A, et al.. 2022. Biosynthetic potential of the global ocean microbiome. Nature 607:111–118. doi:10.1038/s41586-022-04862-3 35732736 PMC9259500

[15] Sharrar AM, Crits-Christoph A, Méheust R, Diamond S, Starr EP, Banfield JF. 2020. Bacterial secondary metabolite biosynthetic potential in soil varies with phylum, depth, and vegetation type. mBio 11:e00416-20. doi:10.1128/mBio.00416-20 32546614 PMC7298704

[16] Zhang Z, Zhang L, Zhang L, Chu H, Zhou J, Ju F. 2024. Diversity and distribution of biosynthetic gene clusters in agricultural soil microbiomes. mSystems 9:e01263-23. doi:10.1128/msystems.01263-23 38470142 PMC11019929

[17] Nayfach S, Roux S, Seshadri R, Udwary D, Varghese N, Schulz F, Wu D, Paez-Espino D, Chen I-M, Huntemann M, et al.. 2021. A genomic catalog of Earth’s microbiomes. Nat Biotechnol 39:499–509. doi:10.1038/s41587-020-0718-6 33169036 PMC8041624

[18] Bağcı C, Nuhamunada M, Goyat H, Ladanyi C, Sehnal L, Blin K, Kautsar SA, Tagirdzhanov A, Gurevich A, Mantri S, von Mering C, Udwary D, Medema MH, Weber T, Ziemert N. 2025. BGC Atlas: a web resource for exploring the global chemical diversity encoded in bacterial genomes. Nucleic Acids Res 53:D618–D624. doi:10.1093/nar/gkae953 39470730 PMC11701567

[19] Wilkie JP, Dye DW, Watson DRW. 1973. Further hosts of Pseudomonas viridiflava. N Z J Agric Res 16:315–323. doi:10.1080/00288233.1973.10421110

[20] Goss EM, Kreitman M, Bergelson J. 2005. Genetic diversity, recombination and cryptic clades in Pseudomonas viridiflava infecting natural populations of Arabidopsis thaliana. Genetics 169:21–35. doi:10.1534/genetics.104.031351 15489535 PMC1448860

[21] Lundberg DS, de Pedro Jové R, Pramoj Na Ayutthaya P, Karasov TL, Shalev O, Poersch K, Ding W, Bollmann-Giolai A, Bezrukov I, Weigel D. 2022. Contrasting patterns of microbial dominance in the Arabidopsis thaliana phyllosphere. Proc Natl Acad Sci USA 119:e2211881119. doi:10.1073/pnas.2211881119 36538480 PMC9907089

[22] Jakob K, Goss EM, Araki H, Van T, Kreitman M, Bergelson J. 2002. Pseudomonas viridiflava and P. syringae—natural pathogens of Arabidopsis thaliana. Mol Plant Microbe Interact 15:1195–1203. doi:10.1094/MPMI.2002.15.12.1195 12481991

[23] Goss EM, Bergelson J. 2007. Fitness consequences of infection of Arabidopsis thaliana with its natural bacterial pathogen Pseudomonas viridiflava. Oecologia 152:71–81. doi:10.1007/s00442-006-0631-9 17180370

[24] Karasov TL, Almario J, Friedemann C, Ding W, Giolai M, Heavens D, Kersten S, Lundberg DS, Neumann M, Regalado J, Neher RA, Kemen E, Weigel D. 2018. Arabidopsis thaliana and Pseudomonas pathogens exhibit stable associations over evolutionary timescales. Cell Host Microbe 24:168–179. doi:10.1016/j.chom.2018.06.011 30001519 PMC6054916

[25] Karasov TL, Neumann M, Leventhal L, Symeonidi E, Shirsekar G, Hawks A, Monroe G, Team P, Exposito-Alonso M, Bergelson J, Weigel D, Schwab R. 2024. Continental-scale associations of Arabidopsis thaliana phyllosphere members with host genotype and drought. Nat Microbiol 9:2748–2758. doi:10.1038/s41564-024-01773-z 39242816 PMC11457713

[26] Shalev O, Karasov TL, Lundberg DS, Ashkenazy H, Pramoj Na Ayutthaya P, Weigel D. 2022. Commensal Pseudomonas strains facilitate protective response against pathogens in the host plant. Nat Ecol Evol 6:383–396. doi:10.1038/s41559-022-01673-7 35210578 PMC8986537

[27] Duque-Jaramillo A, Ulmer N, Alseekh S, Bezrukov I, Fernie AR, Skirycz A, Karasov TL, Weigel D. 2023. The genetic and physiological basis of Arabidopsis thaliana tolerance to Pseudomonas viridiflava. New Phytol 240:1961–1975. doi:10.1111/nph.19241 37667565

[28] Shahid I, Malik KA, Mehnaz S. 2018. A decade of understanding secondary metabolism in Pseudomonas spp. for sustainable agriculture and pharmaceutical applications. Environ Sustain 1:3–17. doi:10.1007/s42398-018-0006-2

[29] Gross H, Loper JE. 2009. Genomics of secondary metabolite production by Pseudomonas spp. Nat Prod Rep 26:1408–1446. doi:10.1039/b817075b 19844639

[30] Alam K, Islam MM, Li C, Sultana S, Zhong L, Shen Q, Yu G, Hao J, Zhang Y, Li R, Li A. 2021. Genome mining of Pseudomonas species: diversity and evolution of metabolic and biosynthetic potential. Molecules 26:7524. doi:10.3390/molecules26247524 34946606 PMC8704066

[31] Gavriilidou A, Kautsar SA, Zaburannyi N, Krug D, Müller R, Medema MH, Ziemert N. 2022. Compendium of specialized metabolite biosynthetic diversity encoded in bacterial genomes. Nat Microbiol 7:726–735. doi:10.1038/s41564-022-01110-2 35505244

[32] Rieusset L, Rey M, Muller D, Vacheron J, Gerin F, Dubost A, Comte G, Prigent-Combaret C. 2020. Secondary metabolites from plant-associated Pseudomonas are overproduced in biofilm. Microb Biotechnol 13:1562–1580. doi:10.1111/1751-7915.13598 33000552 PMC7415375

[33] Bricout A, Morris CE, Chandeysson C, Duban M, Boistel C, Chataigné G, Lecouturier D, Jacques P, Leclère V, Rochex A. 2022. The diversity of lipopeptides in the Pseudomonas syringae complex parallels phylogeny and sheds light on structural diversification during evolutionary history. Microbiol Spectr 10:e01456-22. doi:10.1128/spectrum.01456-22 PMC9769872 36287007

[34] Stringlis IA, Zhang H, Pieterse CMJ, Bolton MD, de Jonge R. 2018. Microbial small molecules – weapons of plant subversion. Nat Prod Rep 35:410–433. doi:10.1039/c7np00062f 29756135

[35] Torres M, Paszti S, Eberl L. 2024. Shedding light on bacteria-host interactions with the aid of TnSeq approaches. mBio 15:e00390-24. doi:10.1128/mbio.00390-24 38722161 PMC11237515

[36] Pacheco-Moreno A, Stefanato FL, Ford JJ, Trippel C, Uszkoreit S, Ferrafiat L, Grenga L, Dickens R, Kelly N, Kingdon AD, Ambrosetti L, Nepogodiev SA, Findlay KC, Cheema J, Trick M, Chandra G, Tomalin G, Malone JG, Truman AW. 2021. Pan-genome analysis identifies intersecting roles for Pseudomonas specialized metabolites in potato pathogen inhibition. eLife 10:e71900. doi:10.7554/eLife.71900 34792466 PMC8719888

[37] Anand A, Falquet L, Abou-Mansour E, L’Haridon F, Keel C, Weisskopf L. 2023. Biological hydrogen cyanide emission globally impacts the physiology of both HCN-emitting and HCN-perceiving Pseudomonas. mBio 14:e00857-23. doi:10.1128/mbio.00857-23 37650608 PMC10653877

[38] Ashkenazy H, Weigel D. 2025. PanGene-O-meter: intra-species diversity based on gene-content. bioRxiv. doi:10.1101/2025.09.01.673544

[39] Navarro-Muñoz JC, Selem-Mojica N, Mullowney MW, Kautsar SA, Tryon JH, Parkinson EI, De Los Santos ELC, Yeong M, Cruz-Morales P, Abubucker S, Roeters A, Lokhorst W, Fernandez-Guerra A, Cappelini LTD, Goering AW, Thomson RJ, Metcalf WW, Kelleher NL, Barona-Gomez F, Medema MH. 2020. A computational framework to explore large-scale biosynthetic diversity. Nat Chem Biol 16:60–68. doi:10.1038/s41589-019-0400-9 31768033 PMC6917865

[40] Zdouc MM, Blin K, Louwen NLL, Navarro J, Loureiro C, Bader CD, Bailey CB, Barra L, Booth TJ, Bozhüyük KAJ, et al.. 2025. MIBiG 4.0: advancing biosynthetic gene cluster curation through global collaboration. Nucleic Acids Res 53:D678–D690. doi:10.1093/nar/gkae1115 39657789 PMC11701617

[41] Backman T, Latorre SM, Symeonidi E, Muszyński A, Bleak E, Eads L, Martinez-Koury PI, Som S, Hawks A, Gloss AD, Belnap DM, Manuel AM, Deutschbauer AM, Bergelson J, Azadi P, Burbano HA, Karasov TL. 2024. A phage tail–like bacteriocin suppresses competitors in metapopulations of pathogenic bacteria. Science 384:eado0713. doi:10.1126/science.ado0713 38870284 PMC11404688

[42] Wetmore KM, Price MN, Waters RJ, Lamson JS, He J, Hoover CA, Blow MJ, Bristow J, Butland G, Arkin AP, Deutschbauer A. 2015. Rapid quantification of mutant fitness in diverse bacteria by sequencing randomly bar-coded transposons. mBio 6:e00306-15. doi:10.1128/mBio.00306-15 25968644 PMC4436071

[43] Love MI, Huber W, Anders S. 2014. Moderated estimation of fold change and dispersion for RNA-seq data with DESeq2. Genome Biol 15:550. doi:10.1186/s13059-014-0550-8 25516281 PMC4302049

[44] Cordero OX, Wildschutte H, Kirkup B, Proehl S, Ngo L, Hussain F, Le Roux F, Mincer T, Polz MF. 2012. Ecological populations of bacteria act as socially cohesive units of antibiotic production and resistance. Science 337:1228–1231. doi:10.1126/science.1219385 22955834

[45] Collins C, Didelot X. 2018. A phylogenetic method to perform genome-wide association studies in microbes that accounts for population structure and recombination. PLoS Comput Biol 14:e1005958. doi:10.1371/journal.pcbi.1005958 29401456 PMC5814097

[46] Lipps SM, Samac DA. 2022. Pseudomonas viridiflava: an internal outsider of the Pseudomonas syringae species complex. Mol Plant Pathol 23:3–15. doi:10.1111/mpp.13133 34463014 PMC8659605

[47] Salamzade R, Kalan LR. 2025. Context matters: assessing the impacts of genomic background and ecology on microbial biosynthetic gene cluster evolution. mSystems 10:e01538-24. doi:10.1128/msystems.01538-24 39992097 PMC11915812

[48] Genomic Standards Consortium. 2025. MIBiG minimum information about a biosynthetic gene cluster. Statistics. Available from: https://mibig.secondarymetabolites.org/stats. Retrieved 5 Jul 2025.

[49] Mukherjee A, Tikariha H, Bandla A, Pavagadhi S, Swarup S. 2023. Global analyses of biosynthetic gene clusters in phytobiomes reveal strong phylogenetic conservation of terpenes and aryl polyenes. mSystems 8:e0038723. doi:10.1128/msystems.00387-23 37409823 PMC10469690

[50] Cimermancic P, Medema MH, Claesen J, Kurita K, Wieland Brown LC, Mavrommatis K, Pati A, Godfrey PA, Koehrsen M, Clardy J, Birren BW, Takano E, Sali A, Linington RG, Fischbach MA. 2014. Insights into secondary metabolism from a global analysis of prokaryotic biosynthetic gene clusters. Cell 158:412–421. doi:10.1016/j.cell.2014.06.034 25036635 PMC4123684

[51] Götze S, Stallforth P. 2020. Structure, properties, and biological functions of nonribosomal lipopeptides from pseudomonads. Nat Prod Rep 37:29–54. doi:10.1039/c9np00022d 31436775

[52] Bartoli C, Berge O, Monteil CL, Guilbaud C, Balestra GM, Varvaro L, Jones C, Dangl JL, Baltrus DA, Sands DC, Morris CE. 2014. The Pseudomonas viridiflava phylogroups in the P. syringae species complex are characterized by genetic variability and phenotypic plasticity of pathogenicity-related traits. Environ Microbiol 16:2301–2315. doi:10.1111/1462-2920.12433 24612372

[53] Qi H, Zhao L, Xu L, Liu C, Cheng H, Han X, Ren Y, Xu C, Yan J, Jiang C, Ma B, Ma Z, Chen Y. 2026. A functional atlas of secondary metabolite biosynthetic gene clusters governing growth, stress adaptation, and pathogenicity in Fusarium graminearum. Crop Health 4:8. doi:10.1007/s44297-026-00070-x 41866646 PMC13006487

[54] Helmann TC, Deutschbauer AM, Lindow SE. 2019. Genome-wide identification of Pseudomonas syringae genes required for fitness during colonization of the leaf surface and apoplast. Proc Natl Acad Sci USA 116:18900–18910. doi:10.1073/pnas.1908858116 31484768 PMC6754560

[55] Helmann TC, Deutschbauer AM, Lindow SE. 2020. Distinctiveness of genes contributing to growth of Pseudomonas syringae in diverse host plant species. PLoS One 15:e0239998. doi:10.1371/journal.pone.0239998 32986776 PMC7521676

[56] Price MN, Wetmore KM, Waters RJ, Callaghan M, Ray J, Liu H, Kuehl JV, Melnyk RA, Lamson JS, Suh Y, Carlson HK, Esquivel Z, Sadeeshkumar H, Chakraborty R, Zane GM, Rubin BE, Wall JD, Visel A, Bristow J, Blow MJ, Arkin AP, Deutschbauer AM. 2018. Mutant phenotypes for thousands of bacterial genes of unknown function. Nature 557:503–509. doi:10.1038/s41586-018-0124-0 29769716

[57] Amrhein A, Zhang M, Hacquard S, Heintz-Buschart A, Wippel K. 2025. Pseudomonas intra-genus competition determines the protective function of synthetic bacterial communities in Arabidopsis thaliana. PLoS Biol 23:e3002882. doi:10.1371/journal.pbio.3002882 40663585 PMC12262851

[58] Andrić S, Rigolet A, Argüelles Arias A, Steels S, Hoff G, Balleux G, Ongena L, Höfte M, Meyer T, Ongena M. 2023. Plant-associated Bacillus mobilizes its secondary metabolites upon perception of the siderophore pyochelin produced by a Pseudomonas competitor. ISME J 17:263–275. doi:10.1038/s41396-022-01337-1 36357782 PMC9860033

[59] Thibault D, Jensen PA, Wood S, Qabar C, Clark S, Shainheit MG, Isberg RR, van Opijnen T. 2019. Droplet Tn-Seq combines microfluidics with Tn-Seq for identifying complex single-cell phenotypes. Nat Commun 10:5729. doi:10.1038/s41467-019-13719-9 31844066 PMC6914776

[60] Schreier JE, Smith CB, Ioerger TR, Moran MA. 2023. A mutant fitness assay identifies bacterial interactions in a model ocean hot spot. Proc Natl Acad Sci USA 120:e2217200120. doi:10.1073/pnas.2217200120 36920927 PMC10041152

[61] Smith P, Schuster M. 2019. Public goods and cheating in microbes. Curr Biol 29:R442–R447. doi:10.1016/j.cub.2019.03.001 31163154

[62] Jiricny N, Diggle SP, West SA, Evans BA, Ballantyne G, Ross-Gillespie A, Griffin AS. 2010. Fitness correlates with the extent of cheating in a bacterium. J Evol Biol 23:738–747. doi:10.1111/j.1420-9101.2010.01939.x 20210835

[63] Dumas Z, Kümmerli R. 2012. Cost of cooperation rules selection for cheats in bacterial metapopulations: cost of cooperation and cheating in bacteria. J Evol Biol 25:473–484. doi: 10.1111/j.1420-9101.2011.02437.x 22168669 10.1111/j.1420-9101.2011.02437.x

[64] Drott MT, Debenport T, Higgins SA, Buckley DH, Milgroom MG. 2019. Fitness cost of aflatoxin production in Aspergillus flavus when competing with soil microbes could maintain balancing selection. mBio 10:e02782-18. doi:10.1128/mBio.02782-18 30782658 PMC6381279

[65] Vogwill T, MacLean RC. 2015. The genetic basis of the fitness costs of antimicrobial resistance: a meta-analysis approach. Evol Appl 8:284–295. doi:10.1111/eva.12202 25861386 PMC4380922

[66] Melnyk AH, Wong A, Kassen R. 2015. The fitness costs of antibiotic resistance mutations. Evol Appl 8:273–283. doi:10.1111/eva.12196 25861385 PMC4380921

[67] D’Souza-Ault MR, Smith LT, Smith GM. 1993. Roles of N-acetylglutaminylglutamine amide and glycine betaine in adaptation of Pseudomonas aeruginosa to osmotic stress. Appl Environ Microbiol 59:473–478. doi:10.1128/aem.59.2.473-478.1993 8434912 PMC202129

[68] Blin K, Kim HU, Medema MH, Weber T. 2019. Recent development of antiSMASH and other computational approaches to mine secondary metabolite biosynthetic gene clusters. Brief Bioinformatics 20:1103–1113. doi:10.1093/bib/bbx146 29112695 PMC6781578

[69] Bai C, Bayona LM, van Wezel GP. 2025. Construction and diversification of natural product biosynthetic gene clusters at high efficiency and accuracy. ACS Synth Biol 14:4574–4585. doi:10.1021/acssynbio.5c00601 41070399 PMC12645565

[70] Wang J, Liu N, Liu M, Huang Y. 2026. Eco-evolutionary dynamics sustain a potent yet rare antibiotic gene cluster in Streptomyces. ISME J 20:wrag060. doi:10.1093/ismejo/wrag060 41848078 PMC13099267

[71] Seppey M, Manni M, Zdobnov EM. 2019. BUSCO: assessing genome assembly and annotation completeness. Methods Mol Biol 1962:227–245. doi:10.1007/978-1-4939-9173-0_14 31020564

[72] Jaccard P. 1912. The distribution of the flora in the alpine zone. New Phytol 11:37–50. doi:10.1111/j.1469-8137.1912.tb05611.x

[73] Reitz ZL. 2024. Szreitz/multismash. https://zenodo.org/records/10467162.

[74] Letunic I, Bork P. 2021. Interactive Tree Of Life (iTOL) v5: an online tool for phylogenetic tree display and annotation. Nucleic Acids Res 49:W293–W296. doi:10.1093/nar/gkab301 33885785 PMC8265157

[75] R Core Team. 2020. R: a language and environment for statistical computing. Vienna, Austria. R Foundation for Statistical Computing

[76] Brereton RG. 2021. Empirical and statistical p values and Type 1 error rates: Putting it all together. J Chemom 35:e3330. doi:10.1002/cem.3330

