# Supplementary material for: Biosynthetic gene clusters in Pseudomonas viridiflava have a fitness cost during Arabidopsis thaliana infection
Source: mSystems. 2026 Jun 15;11(7):e00212-26. doi: 10.1128/msystems.00212-26 (PMC13387001; doi:10.1128/msystems.00212-26)

Supplementary material for:

Biosynthetic gene clusters in *Pseudomonas viridiflava* have a fitness cost during *Arabidopsis thaliana* infection

Alejandra Duque-Jaramillo,[a] Efthymia Symeonidi,[b] Manuela Neumann,[a,*] Haim Ashkenazy,[a] Madelyn Allen,[b] Detlef Weigel,[a,c]# Talia L. Karasov[a,b]#

[a]Department of Molecular Biology, Max Planck Institute for Biology Tübingen, 72076 Tübingen, Germany

[b]School of Biological Sciences, University of Utah, Salt Lake City, UT 84112, USA

[c]Institute for Bioinformatics and Medical Informatics, University Tübingen, 72076 Tübingen, Germany

Running title: BGC fitness costs in *Arabidopsis*-associated *Pseudomonas*

#Address correspondence to Detlef Weigel, weigel@tue.mpg.de, and Talia Karasov, t.karasov@utah.edu

*Present address: Robert Bosch GmbH, Corporate Sector Research and Advance Engineering, 71272 Renningen, Germany

In this document: Figures S1 to S5

Tables S1 to S5 are provided in an additional Excel file

Figure S1 - Relation between genome assembly statistics and BGC prediction. The number of predicted BGCs vs the number of contigs in a genome (A) and the genome's completeness as per CheckM (B). Proportion of predicted BGCs located in a contig edge vs the number of contigs in a genome (C) and the genome's completeness as per CheckM (D). Triangles indicate strains for which disease severity data was available.

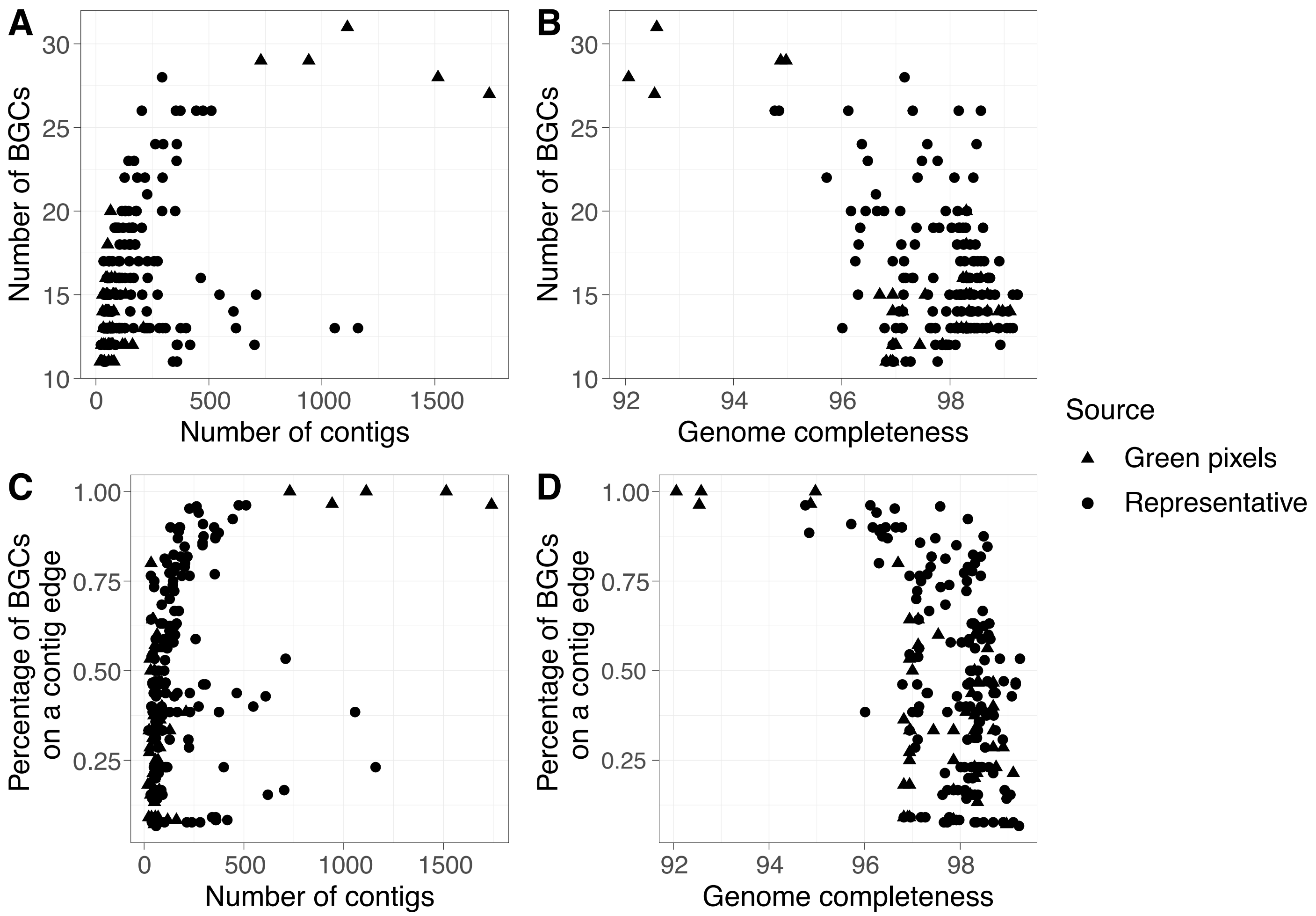

Figure S2 - Presence/abscence matrix for all GCFs predicted in *P. viridiflava* isolates. Maximum likelihood phylogenetic tree of 225 *P. viridiflava* isolates based on 939 concatenated core genes and presence (shades of gray) or absence (white) of gene cluster families (GCFs). The first column indicates the inclusion criteria for each isolate: either due to being a representative genome (blue) or due to having disease data on *A. thaliana* available (orange). Purple indicates isolate p25.C2, the background in which the RB-Tn-Seq mutant library used in this study was generated.

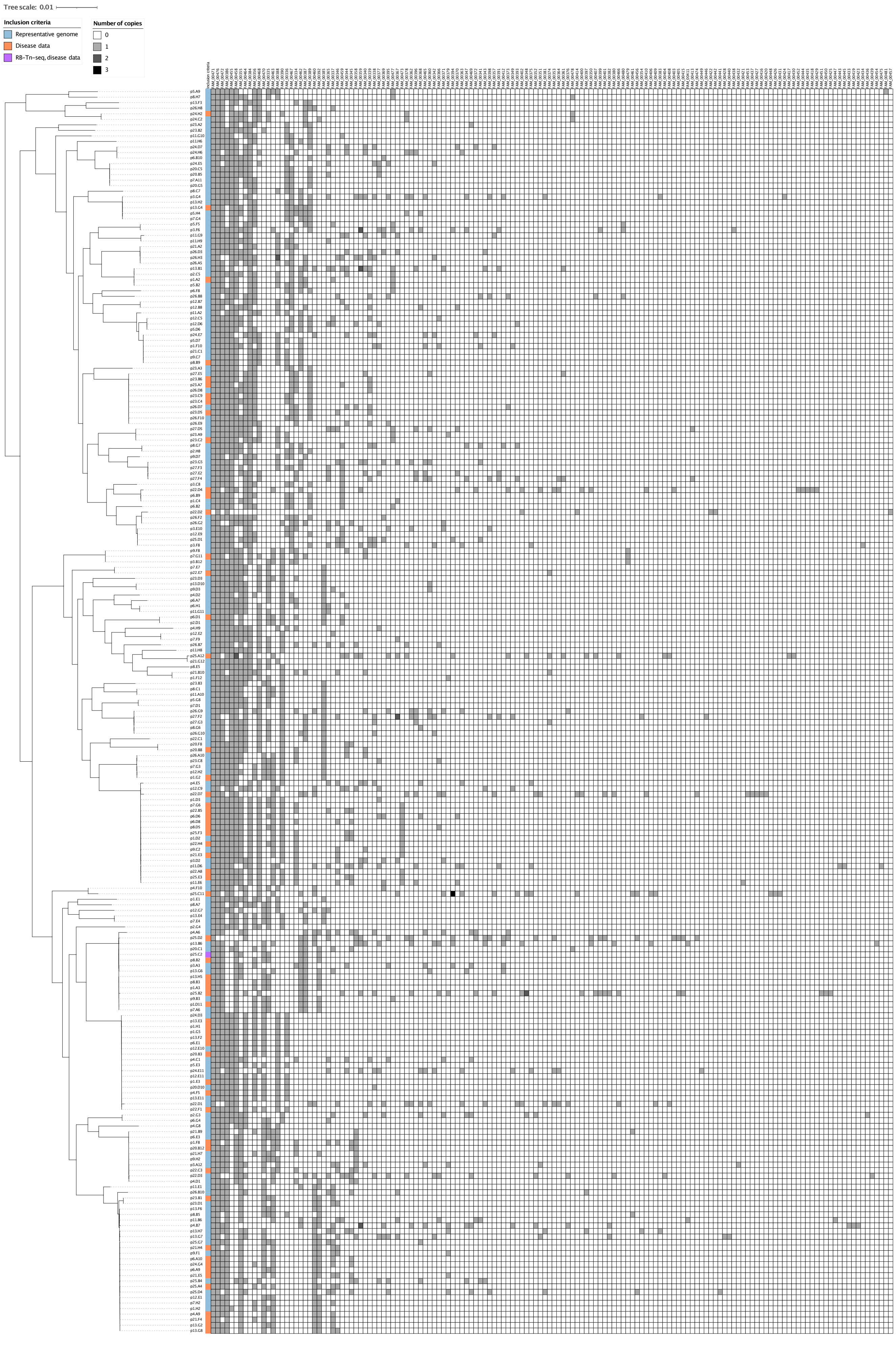
Tree scale: 0.01
Inclusion criteria
Representative genome
Disease data
RB–Tn–seq, disease data
Number of copies
0
1
2
3

Figure S3 - BGCs predicted in *P. viridiflava* ATUE5:p25.C2. Diagram of the eight regions encoding 11 BGCs predicted by antiSMASH. Each arrow represents a gene and the color of the arrow indicates the gene function. There were no BGCs on contigs' edges. Asterisks on top of each BGC diagram indicate genes for which DAMs were identified in hosts Ey15-2 (red) and Col-0 (black).

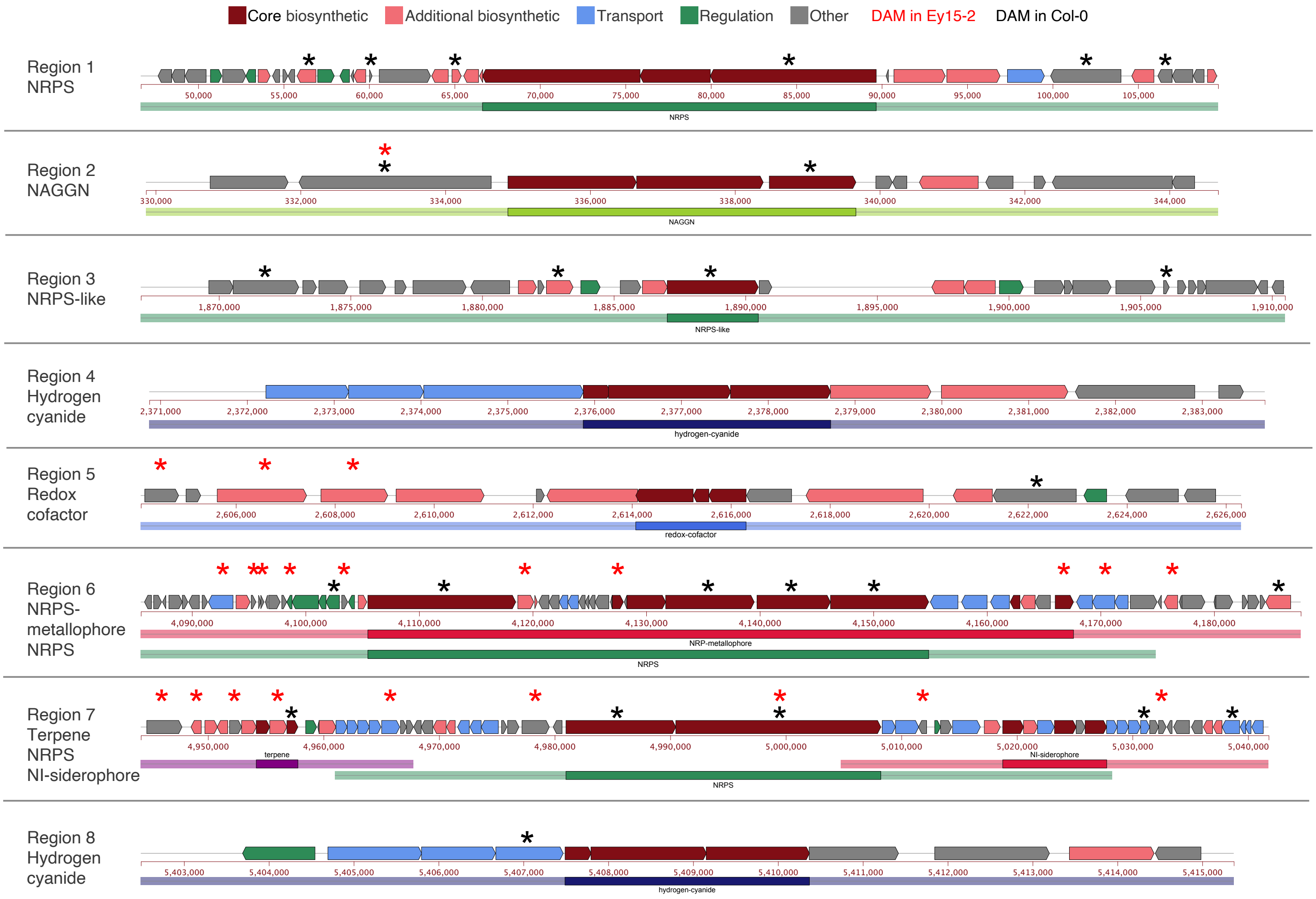

Figure S4 - Distribution of abundance changes in the p25.C2 mutant pool. For all or a subset of BGC-associated mutants in hosts Ey15-2 (A) and Col-0 (B).

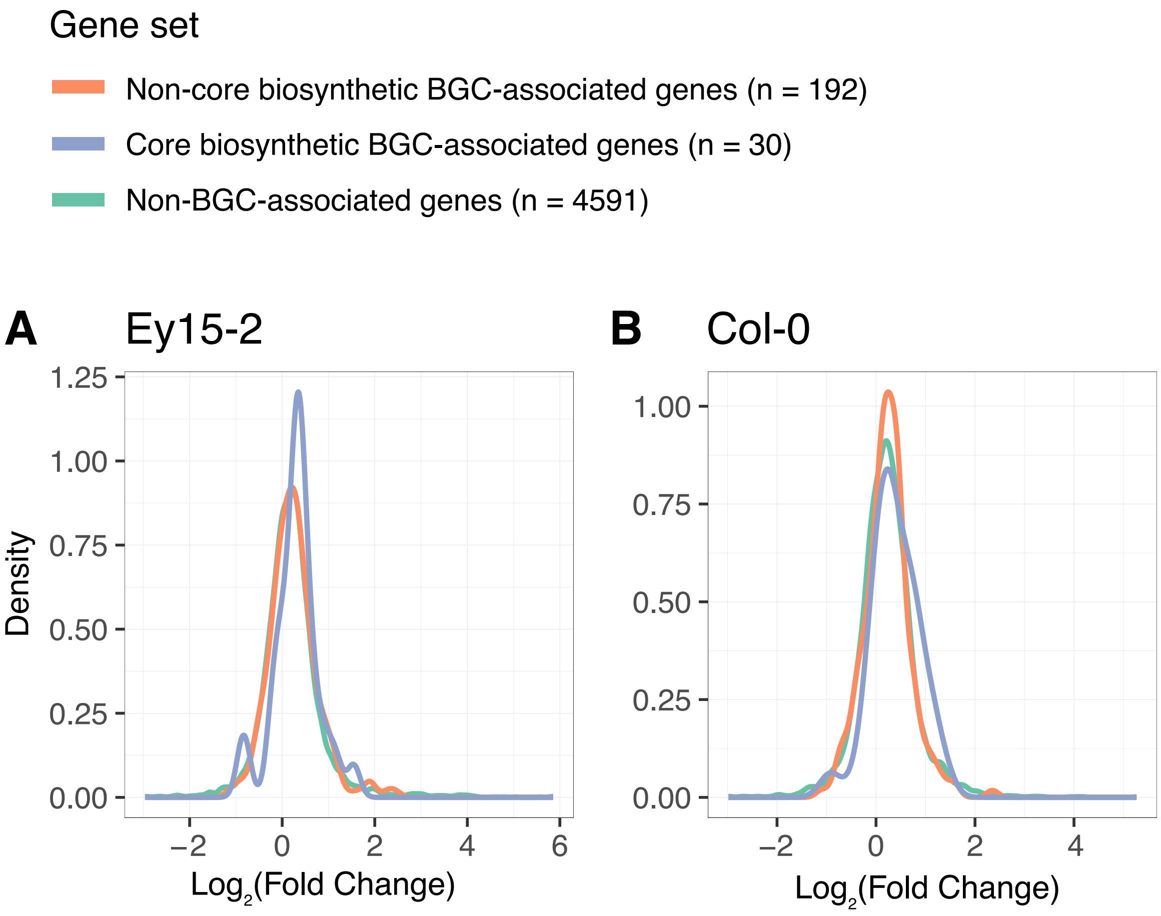

Figure S5 - Volcano plot of p25.C2 mutant pool in LB medium. Abundance change (abundance after 24 hours of growth in LB media compared to inoculum) of the BarSeq p25.C2 mutant pool. A total of 4,156 genes had mutants, 198 of which were BGC-associated genes (orange points). Dashed lines indicate no change in abundance (x-axis, $\log_2$ fold change = 0) and the significance threshold (y-axis, p = 0.05).

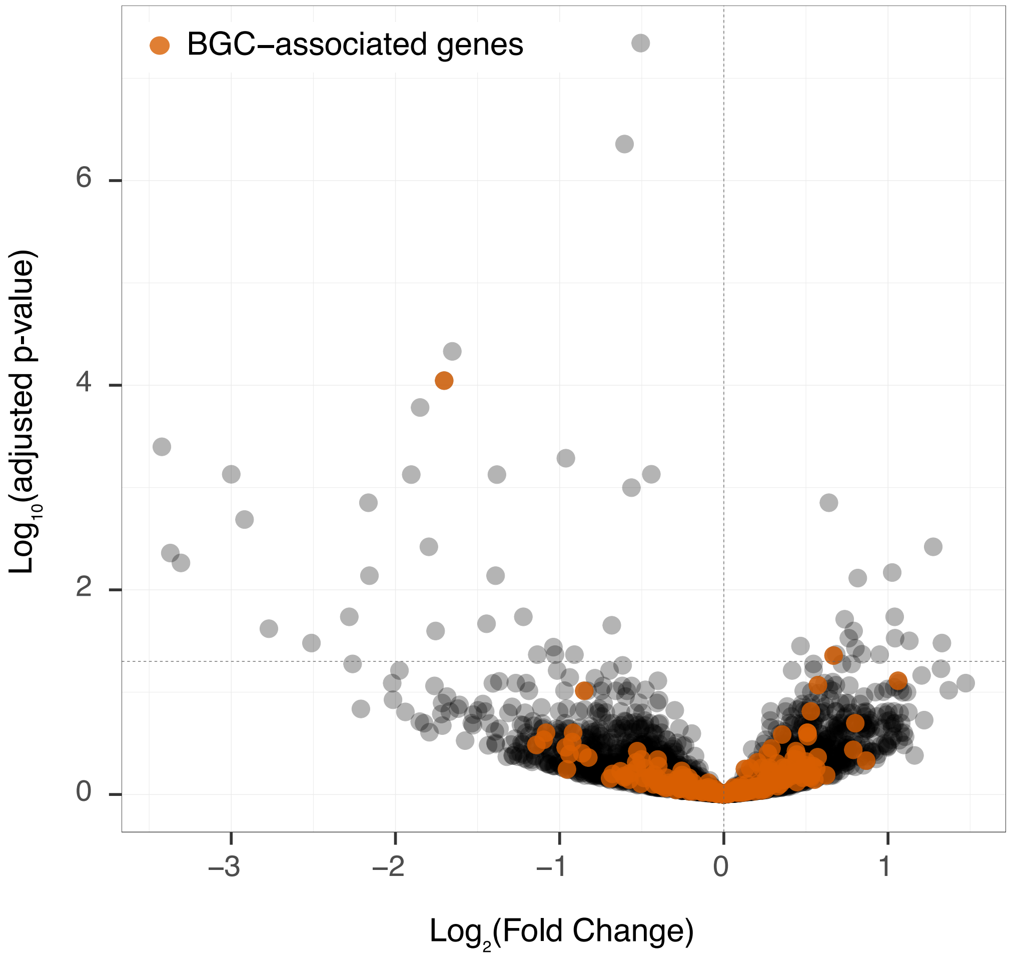

Supplement: Supplemental figures — Figures S1 to S5. [file msystems.00212-26-s0001.pdf]
