# Supplementary material for: Biosynthetic gene clusters in Pseudomonas viridiflava have a fitness cost during Arabidopsis thaliana infection
Source: mSystems. 2026 Jun 15;11(7):e00212-26. doi: 10.1128/msystems.00212-26 (PMC13387001; doi:10.1128/msystems.00212-26)
Supplement: Reviewer comments [file reviewer-comments.pdf]

AMERICAN SOCIETY FOR MICROBIOLOGY | mSystems®

Biosynthetic gene clusters in *Pseudomonas viridiflava* have a fitness cost during *Arabidopsis thaliana* infection

Alejandra Duque-Jaramillo, Efthymia Symeonidi, Manuela Neumann, Haim Ashkenazy, Madelyn Allen, Detlef Weigel, and Talia Karasov

Corresponding Author(s): Talia Karasov, The University of Utah School of Biological Sciences

Review Timeline:

Submission Date:	February 18, 2026
Editorial Decision:	March 11, 2026
Revision Received:	May 1, 2026
Accepted:	May 10, 2026

Editor: Ákos T. Kovács

Reviewer(s): The reviewers have opted to remain anonymous.

Transaction Report:

(Note: With the exception of the correction of typographical or spelling errors that could be a source of ambiguity, letters and reports are not edited. The original formatting of letters and referee reports may not be reflected in this compilation.)

DOI: https://doi.org/10.1128/msystems.00212-26

Re: mSystems00212-26 (**Biosynthetic gene clusters in *Pseudomonas viridiflava* have a fitness cost during *Arabidopsis thaliana* infection**)

Dear Prof. Talia L Karasov:

Thank you for the privilege of reviewing your work. Below you will find my comments, instructions from the mSystems editorial office, and the reviewer comments.

Your manuscript was commented by two reviewers who suggest several clarification in your manuscript that should be addressed in your revision. I agree with Reviewer 1 that testing fitness in standard laboratory culturing conditions could also reveal the cost of carrying BGCs, which should be addressed either experimentally or presenting previous evidence related to the Pseudomonas species used in the study. Importantly, following the reviewers' suggestions during your revision should improve the biological rooting in the systems microbiology side of the work.

Please return the manuscript within 60 days; if you cannot complete the modification within this time period, please contact me. If you do not wish to modify the manuscript and prefer to submit it to another journal, notify me immediately so that the manuscript may be formally withdrawn from consideration by mSystems.

Revision Guidelines
To submit your modified manuscript, log into the submission site at https://msystems.msubmit.net/cgi-bin/main.plex. Go to Author Tasks and click the appropriate manuscript title to begin. The information you entered when you first submitted the paper will be displayed; update this as necessary. Note the following requirements:

• Upload point-by-point responses to the issues raised by the reviewers in a file named "Response to Reviewers," NOT in your cover letter.
• Upload a compare copy of the manuscript (without figures) as a "Marked-Up Manuscript" file.
• Upload a clean .DOC/.DOCX version of the revised manuscript and remove the previous version.
• Each figure must be uploaded as a separate, editable, high-resolution file (TIFF or EPS preferred), and any multipanel figures must be assembled into one file.
• Any supplemental material intended for posting by ASM should be uploaded with their legends separate from the main manuscript. You can combine all supplemental material into one file (preferred) or split it into a maximum of 10 files with all associated legends included.

For complete guidelines on revision requirements, see our Submission and Review Process webpage. Submission of a paper that does not conform to guidelines may delay acceptance of your manuscript.

Data availability: ASM policy requires that data be available to the public upon online posting of the article, so please verify all links to sequence records, if present, and make sure that each number retrieves the full record of the data. If a new accession number is not linked or a link is broken, provide mSystems production staff with the correct URL for the record. If the accession numbers for new data are not publicly accessible before the expected online posting of the article, publication may be delayed; please contact production staff (mSystems@asmusa.org) immediately with the expected release date.

Publication Fees: For information on publication fees and which article types are subject to charges, visit our website. If your manuscript is accepted for publication and any fees apply, you will be contacted separately about payment during the production process; please follow the instructions in that e-mail. Arrangements for payment must be made before your article is published.

ASM Membership: Corresponding authors may join or renew ASM membership to obtain discounts on publication fees. Need to upgrade your membership level? Please contact Customer Service at Service@asmusa.org.

The ASM Journals program strives for constant improvement in our submission and publication process. Please tell us how we can improve your experience by taking this quick Author Survey.

Thank you for submitting your paper to mSystems.

Sincerely,
Ákos T. Kovács
Editor
mSystems

Reviewer #1 (Comments for the Author):

This study combined in vivo and in vitro experiments to assess the fitness costs of BGCs during A. thaliana infection. Contrary to the initial assumption, they found that BGC-encoded specialized metabolites appear to play a limited role in the virulence of P. viridiflava on A. thaliana. This study provides new evidence regarding the fitness costs associated with BGCs in plant-associated environments. However, I have several issues need to be clarified.

Major comments:
The authors used two A. thaliana environments to validate mutant-fitness correlations. Were these BGCs also costly under standard laboratory culturing conditions (e.g., rich media)? If these BGCs impose a substantial metabolic burden, mutants might exhibit increased fitness across multiple environments, not only during plant infection. Testing this possibility would help determine whether the observed fitness effects are plant-specific or more general.

Why did the authors expect BGC presence to be positively correlated with plant infection? BGCs are metabolically expensive, and even if some BGCs contribute to pathogenicity, it is more common that only a subset of clusters is directly involved. The authors mention that the abundance of only two and four mutants was reduced in Col-0 and Ey15-2 (line 299). Could these specific gene clusters represent key virulence determinants? This point requires further discussion.

Authors found that NRPS clusters are among the most commonly characterized BGCs in Pseudomonas and other bacteria. Are the findings here specific to P. viridiflava ATUE5 genomes, or do they reflect a broader pattern observed in other Pseudomonas species? The authors should clarify the extent to which their results are species- or lineage-specific.

Other comments:
In the abstract, quantitative results should be provided instead of only descriptive statements. In addition, if particular gene clusters contribute to pathogenicity, they should be explicitly mentioned.

L53: Does P. viridiflava reside on plant leaves in this study? Please clarify the ecological context.

L112: If the stated hypothesis was not supported by the results, the sentence should be rephrased accordingly.

L279: The word "surprised" is not appropriate without prior supporting data. Given that BGCs are metabolically costly and not necessarily directly linked to pathogenicity, the observed result may not be unexpected. Please revise.

L339: not clear.

L437: Ref can be found to support this result I believe.

Figure 4: B and C not clear. Should be noted in the figure instead of the Figure legend.

L566: What are the control groups in the plant infection experiments? For example, was there a medium-only control without plants?

L509: "-if any -upon"?

577: The statistical methods require appropriate references.

Reviewer #2 (Comments for the Author):

The study by Duque-Jaramillo represents an important and systematic analysis of the secondary metabolic potential within populations of Pseudomonas viridiflava. The results highlight a large number of uncharacterized BGCs. The study also aims to assess the contribution of this biosynthetic potential to direct interactions with plants. However, several central concepts related to the phylogenetic analysis and the assessment and association of BGCs with in planta fitness require further clarification, as these may affect the conclusion that BGCs impose a fitness cost during plant interactions, as suggested by the title and abstract. My main comments are outlined below:

Main comments

Comment #1: Phylogeny and distributions of BGCs.

From the phylogenetic tree presented in Figure 1, it appears as if there are several large groups of strains that are identical or near-identical. For example, the group from strain p13-G8 to p13-F6. Another example is the group from p11-E6 to p4-E5.
Q1: What is the reason that there is this over-representation of certain groups (sub-lineages) with limited genetic diversity in the phylogenetic tree? It does not appear that it can be explained by your inclusion of 62 ATUE5 isolates for which disease severity

data was available. Or can it? Please comment.

Q2: following up on the question above: I suggest to include a description and interpretation of the observed phylogenetic relationship among the selected genomes.

Q3: Focusing on the group of strains from p11-E6 to p4-E5: In this group of near-identical strains, most of them appear - perhaps as expected(?) - to have similar content of BGCs. Except for p22-D7 which appear to have a higher content of BGC. There are many of this type of examples in the phylogenetic tree. Here are some of them: Strain p25-B2 has a high number of BGCs but the strain is part of a group of near-identical strains that have a relatively low number of BGCs. Also: Strain p25-A12 has many BGCs but its most closely related strain (p21-G12) has a low number. Several additional examples. What could the possible explanations to these unexpected BGC distributions be? Horisontal gene transfer? Strain or lineage specific gene/BGC acquisition or loss? Or could it be due to sequence quality or other technical challenges? Please comment.

Q4: Following up on Q3: For the examples mentioned above (and the additional examples visible in the figure) it appears that there is a loss of correlation between BGC content and genome phylogeny. Does this influence your examination (and conclusions) of the correlation between "BGC" presence and disease?

Q5: I suggest to use a label in Figure 1 so that both the representative strains and the "disease" strains are identifiable. Perhaps also the p25.C2 strain.

Comment #2: Assessments of the fitness benefits/costs of BGC

Q6: There are examples of regions where some of the mutants have a positive fitness impact while others have a negative - For example regions 5 and 6 in Ey15-2 (figure 3ab). Can you speculate how this can happen?

Q7: I think the reader may be interested in knowing which particular mutants had neutral, negative or positive effects. In other words, having access to the details which is plotted in Figure 3. Perhaps in an excel sheet. This information may become relevant for others that may work on these regions in the future.

Q8: I would argue that it is important to differentiate more clearly between "BGCs" and "BGC-associated genes" in your text (including in title and abstract). As I read your results, your analysis of the 11 BGC show that "only" one of them (region 6) contribute to fitness (and only in Ey15-2) while the remaining 10 do not (Table 2)! On the other hand, you do indeed find that about 10% of the total number of genes that are "associated" with (within) these 11 BGC do have fitness effects. But for many (if not most) of these genes it is unclear if they have any role in the "function" of the BGC. The boundaries and length of the BGC are somewhat arbitrary defined and the genes included in these clusters may in fact have nothing to do with the production of secondary metabolites. So I suggest to use "BGC-associated genes". In short: I am not convinced that finding one or more fitness-modulating 'BGC-associated genes' in most of the 11 BGCs is sufficient to conclude that the majority of BGCs affect fitness. Please clarify

Prof. Ákos T. Kovács
Editor
mSystems

Dear Prof. Kovács,

Thank you for the opportunity to revise and resubmit our manuscript "Biosynthetic gene clusters in *Pseudomonas viridiflava* have a fitness cost during *Arabidopsis thaliana* infection" (mSystems00212-26).

We are grateful for the positive feedback and comments from the reviewers, which we have diligently addressed. The suggested changes substantially improved the clarity of our results and strengthened our conclusions. Here we enclose our point-by-point response to the reviewers' comments and suggestions. Please note that the line numbers refer to the updated manuscript document without tracked changes.

We look forward to your positive response to the revised version of the manuscript.

Best regards,

Talia Karasov and Detlef Weigel

Reviewer #1 (Comments for the Author):

This study combined in vivo and in vitro experiments to assess the fitness costs of BGCs during A. thaliana infection. Contrary to the initial assumption, they found that BGC-encoded specialized metabolites appear to play a limited role in the virulence of P. viridiflava on A. thaliana. This study provides new evidence regarding the fitness costs associated with BGCs in plant-associated environments. However, I have several issues need to be clarified.

Major comments:
The authors used two A. thaliana environments to validate mutant-fitness correlations. Were these BGCs also costly under standard laboratory culturing conditions (e.g., rich

media)? If these BGCs impose a substantial metabolic burden, mutants might exhibit increased fitness across multiple environments, not only during plant infection. Testing this possibility would help determine whether the observed fitness effects are plant-specific or more general.

Thank you for this suggestion that has made our conclusions stronger. We have added results on the cost of BGCs *in vitro* (lines 273-282). We grew the BarSeq mutant pool in LB media and compared the abundance of the mutants after 24 hours of growth *vs.* the inoculum used for all experiments *i.e.* before any growth. The distribution of the fold change of BGC-associated genes was not significantly different from that of non-BGC-genes. In addition, only two BGC-associated genes had a significantly different expression 24 hours after growth in rich media, corresponding to 1% of BGC-associated genes; while we identified >20 differentially abundant mutants in each Col-0 and Ey15-2 *A. thaliana* genotypes (~10% of BGC-associated genes). These results, together with our finding that fitness costs are not consistent across the two *A. thaliana* genotypes tested, with some even having different directions (Fig. 3) suggests that at least the majority of fitness effects we observed are plant-specific.

Why did the authors expect BGC presence to be positively correlated with plant infection? BGCs are metabolically expensive, and even if some BGCs contribute to pathogenicity, it is more common that only a subset of clusters is directly involved.

We agree with the reviewer that BGCs are metabolically expensive and that the expectation is that only a subset of BGC clusters would be directly involved in the virulence or the disease-causing ability of *Pseudomonas viridiflava* isolates. It is not clear what the general role of BGC is in virulence, if any, beyond well-characterized virulence factors such as toxins and siderophores, and this is what we set out to answer in the work we present in this manuscript. We expected that encoding at least some BGCs would contribute/increase virulence of *P. viridiflava in planta*, suggesting their product is as virulence factors. Hence, we expected to see a fitness cost *in planta* for (some) BGC mutants but instead found that, in general, mutants grew better *in planta*, i.e., a general fitness benefit to mutating a given BGC. We have better defined our driving question in the introduction (Lines 114-118).

The authors mention that the abundance of only two and four mutants was reduced in Col-0 and Ey15-2 (line 299). Could these specific gene clusters represent key virulence determinants? This point requires further discussion.

The mutants with a significant negative fold change were for genes located in regions 5 (both in Col-0 and Ey15-2), region 6 (only in Ey15-2) and region 7 (only in Col-0). This information is now easily accessible via tables S4 and S5.

Our results suggest that regions 6 and 7 are not key virulence determinants. The presence of region 6 was positively correlated with the number of green pixels in our data (FAM_00355), indicating that *P. viridiflava* isolates encoding this BGC cause less severe disease in *A. thaliana*. None of the three GCFs constituting region 7 was significant in this correlation analysis (FAM_00335, FAM_00458, FAM_00462).

On the other hand, region 5, encoding a redox cofactor could be a key virulence determinant. Although only a few DAMs were identified in this region, most (75%) of them had a negative fold change, which was not the case for other regions (Fig 3A,B), and mutants with a negative fold change were identified in both host genotypes. Due to the high prevalence of the corresponding GCF (FAM_00476), found in 74 of the 75 isolates with green pixels data, we could not test the correlation between encoding this BGC and the disease severity *in planta*. Additional experiments are required to conclude about the role of this BGC and its product on *A. thaliana* infection.

We have added all these points to the results and discussion, which improves the framing and relevance of our results (lines 315-327, 465-474).

Authors found that NRPS clusters are among the most commonly characterized BGCs in Pseudomonas and other bacteria. Are the findings here specific to P. viridiflava ATUE5 genomes, or do they reflect a broader pattern observed in other Pseudomonas species? The authors should clarify the extent to which their results are species- or lineage-specific.

We found that NRPS are the most common BGCs class in our collection of *P. viridiflava*. This is in line with previous findings of the *Pseudomonas* genus, as we indicate in the discussion (line 379). On the contrary, we did not identify

PKS nor PKS/NRPS hybrids, which have been described in *Pseudomonas* before (lines 375-378).

Although all isolates included in this study were isolated in Southwest Germany, it is important to note that we have isolated *P. viridiflava* ATUE5 across Europe (1) and that all of our isolates with a NCBI RefSeq assembly identifier (GCF_XXXX, 221/225 isolates) are classified as *Pseudomonas_E viridiflava* according to the Genome Taxonomy Database (GTDB)(2). We believe the characterization of the BGC repertoire we present extends to other plant-associated *P. viridiflava*, while the fitness effects are pathosystem-specific (i.e. *P. viridiflava* infection of *A. thaliana)*, as we did not test other hosts or microbe-microbe interactions.

Other comments:
In the abstract, quantitative results should be provided instead of only descriptive statements. In addition, if particular gene clusters contribute to pathogenicity, they should be explicitly mentioned.

We have modified the abstract according to the reviewer's suggestion (lines 37, 43-46).

L53: Does P. viridiflava reside on plant leaves in this study? Please clarify the ecological context.

Yes, *P. viridiflava* resides on plant leaves in this study; it is a leaf pathogen. We indicate this both in the importance and introduction sections (lines 57 and 97). We have modified the first sentence of the Importance to introduce the ecological context of *P. viridiflava* earlier, as follows: "Bacteria, including plant-associated bacteria such as *Pseudomonas viridiflava*, produce a vast array of chemical compounds" (line 52).

L112: If the stated hypothesis was not supported by the results, the sentence should be rephrased accordingly.

The stated hypothesis was our starting hypothesis but it was indeed not supported by our results. We have modified this phrase to "we investigated whether the specialized metabolites encoded by *A. thaliana*-associated *P.*

viridiflava are involved in infection and disease severity on this host" (lines 116-118).

L279: The word "surprised" is not appropriate without prior supporting data. Given that BGCs are metabolically costly and not necessarily directly linked to pathogenicity, the observed result may not be unexpected. Please revise.

We have removed "surprised" and modified the sentence to "We found that the abundance of most BGC mutants increased *in planta*" (line 260).

L339: not clear.

Thank you for pointing out that these results were not clear. We re-wrote the sentence and integrated it into a new paragraph, also in response to some of the major comments above (lines 273-283 and subsequent).

L437: Ref can be found to support this result I believe.

We have added a couple of sentences referencing published work where, in line with our results, encoding a BGC lacked a fitness benefit or resulted in a fitness cost of plant pathogens *in planta* (lines 416-426):

"Despite it being generally stated that BGCs and their products provide a fitness advantage, this is not always the case. In line with our results showing a fitness cost of BGCs for growth *in planta*, almost 90% of BGC mutants in the plant pathogenic fungi *Fusarium graminearum* retained wild-type virulence on wheat (Qi et al. 2026). Mutation of some BGC resulted in bigger lesions in wheat coleoptiles, but this was not statistically significant. In addition, it has been reported that toxin syringomycin was required for competitive fitness of *P. syringae* pv. *syringae* B728a in the apoplast of common bean *Phaseolus vulgaris*, while mutatans in the syringopeptin and syringolin toxins did not have significant apoplastic fitness defects (3). Mutants in phytotoxin synthesis and transport genes even had higher fitness than the wild-type strain in at least one of three plant hosts (common bean, lima bean and pepper) in a host-specific manner (4)."

Figure 4: B and C not clear. Should be noted in the figure instead of the Figure legend.

We were not sure what the reviewer was referring to with this comment and could unfortunately not address it.

L566: What are the control groups in the plant infection experiments? For example, was there a medium-only control without plants?

For the plant infections, the bacterial suspension of pooled mutants was resuspended in $MgSO_4$ and brought to an OD_{600} of 0.02. As a negative control, plants were infected with $MgSO_4$ buffer alone. These mock-infected plants were sequenced as well. We have included this information in the corresponding methods section (line 580).

L509: "-if any -upon"?

We have removed "-if any-" and modified slightly for clarity: "Future experiments should include the characterization of the specialized metabolites produced by *P. viridiflava* isolates and determine their production upon plant infection" (line 518).

577: The statistical methods require appropriate references.

Thank you for pointing this out. We added (5) as a reference for the empirical p-value (line 596).

Reviewer #2 (Comments for the Author):

The study by Duque-Jaramillo represents an important and systematic analysis of the secondary metabolic potential within populations of Pseudomonas viridiflava. The results highlight a large number of uncharacterized BGCs. The study also aims to assess the contribution of this biosynthetic potential to direct interactions with plants. However, several central concepts related to the phylogenetic analysis and the assessment and association of BGCs with in planta fitness require further clarification, as these may affect the conclusion that BGCs impose a fitness cost during plant

interactions, as suggested by the title and abstract. My main comments are outlined below:

Main comments

Comment #1: Phylogeny and distributions of BGCs.

From the phylogenetic tree presented in Figure 1, it appears as if there are several large groups of strains that are identical or near-identical. For example, the group from strain p13-G8 to p13-F6. Another example is the group from p11-E6 to p4-E5.

Q1: What is the reason that there is this over-representation of certain groups (sub-lineages) with limited genetic diversity in the phylogenetic tree? It does not appear that it can be explained by your inclusion of 62 ATUE5 isolates for which disease severity data was available. Or can it? Please comment.

> The overrepresentation of specific groups is because the phylogenetic tree on Fig. 1 was built from the core genome (939 genes from ± 5200 genes in *P. viridiflava* (6), while the selection of representative strains was done based on gene content of both core and accessory genes, which does not necessarily reflect phylogenetic relationships between strains due to e.g. horizontal gene transfer. Strains that are closely related based on their core genome may differ significantly in their accessory genome, which commonly includes BGCs (7); and BGCs and defense mechanisms encoded in mobile elements can be uncoupled from other genomics features (8, 9). Inferring the genetic diversity of a lineage based on a core genome phylogenetic tree can be misleading, as it is the case in our study. For examples, see figure 3 of Ashkenazy and Weigel, 2025, where maximum-likelihood phylogenetic trees based on core-genome and gene-content are compared (10).
>
> To make these key differences more explicit, we have added the number of genes used to build the phylogeny to Figure 1's legend and explicitly stated that we selected representative genomes based on gene content similarity (lines 134, 527-528). We have also included a new supplementary figure, Fig. S4 with the GCFs presence/abscence matrix (see answer to Q3 below for more details).

Q2: following up on the question above: I suggest to include a description and interpretation of the observed phylogenetic relationship among the selected genomes.

> We have added a description and interpretation of the phylogenetic tree in Fig. 1: "Strains with disease data were interleaved with representative genomes and were not evenly distributed across the phylogeny. Clusters of closely related genomes (based on 939 concatenated core genes) were observed, particularly within clades containing several strains included due to the availability of disease data, and irrespective of their similarity to other genomes. However, genomes in these clusters differed in their BGC content, suggesting at least partial decoupling between core genome phylogeny and BGC repertoire. This is consistent with BGCs being common in the accessory genome, often mobile and important for ecological adaptation. Genomes harboring a high number of BGCs were distributed throughout the phylogenetic tree." (lines 159-168).

Q3: Focusing on the group of strains from p11-E6 to p4-E5: In this group of near-identical strains, most of them appear - perhaps as expected(?) - to have similar content of BGCs. Except for p22-D7 which appear to have a higher content of BGC. There are many of this type of examples in the phylogenetic tree. Here are some of them: Strain p25-B2 has a high number of BGCs but the strain is part of a group of near-identical strains that have a relatively low number of BGCs. Also: Strain p25-A12 has many BGCs but its most closely related strain (p21-G12) has a low number. Several additional examples. What could the possible explanations to these unexpected BGC distributions be? Horisontal gene transfer? Strain or lineage specific gene/BGC acquisition or loss? Or could it be due to sequence quality or other technical challenges? Please comment.

> As mentioned in the answer to Q1, the tree in Fig. 1 is a core genome phylogeny, which includes only around 20% of an isolate's genes, while BGCs are most commonly part of the accessory genome instead of the core genome (7). Instances of isolates being closely related based on their core genome but having different BGCs repertoires can indeed be due to horizontal gene transfer and/or gene loss.

It is important also to note that the color strips on Fig. 1 indicate the number of BGCs of a certain class, but not which product this BGC might encode. Even when closely related strains in the phylogeny all encode one arylpolyene, for example, this does not mean they encode the same specialized metabolite. This is why further clustering of the predicted BGC in a set of isolates using e.g., BiG-SCAPE (11) is important, to identify BGCs that are functionally related, i.e., that are likely to produce the same or very similar specialized metabolites.

Furthermore, the isolates indicated by the reviewer are usually isolates that were included due to the availability of disease severity data. We are aware that some of these genomes have lower quality on average, which could affect BGC prediction. We already presented genome quality data on Figure S2 (previously Figure S1), but have now modified it to show the 'source' of genomes, either representatives or with disease severity data.

In addition, we have included a new supplementary figure (Figure S2), which shows the presence/abscence matrix of the 148 GCFs for all isolates along the core phylogenetic tree of Fig. 1. We believe these changes, together with the label suggested in Q5 below, improve clarity about the genomes included in our study and the distribution pattern of BGCs and their relationship with the core gene phylogeny in Fig. 1.

Modified Figure S1

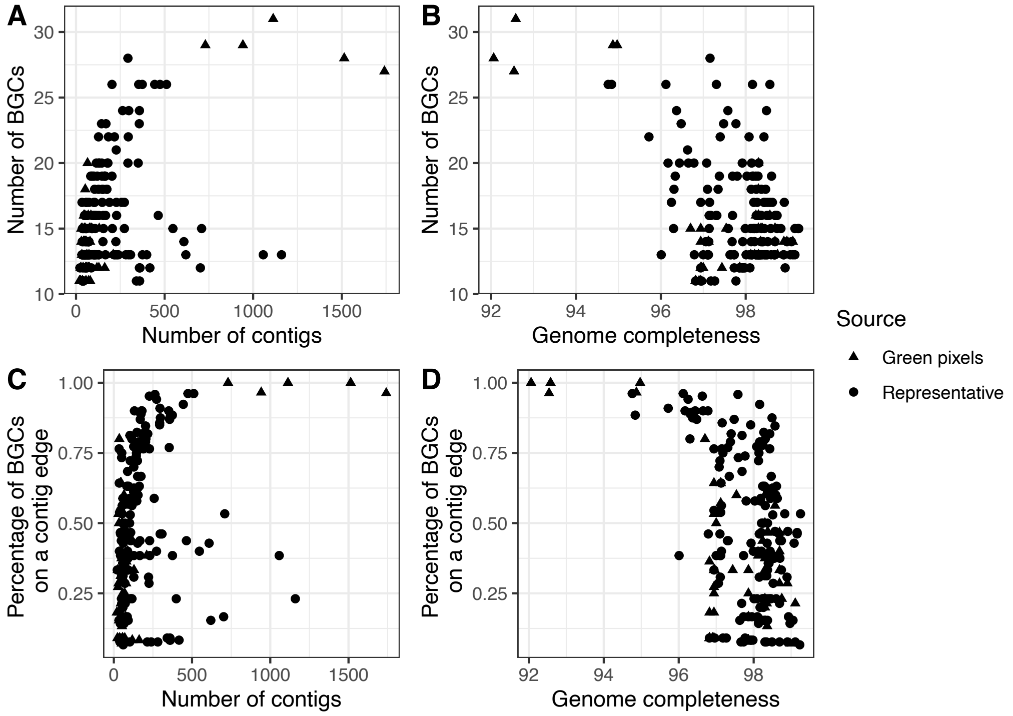

New Figure S2 (only partially displayed here for reference due to large size)

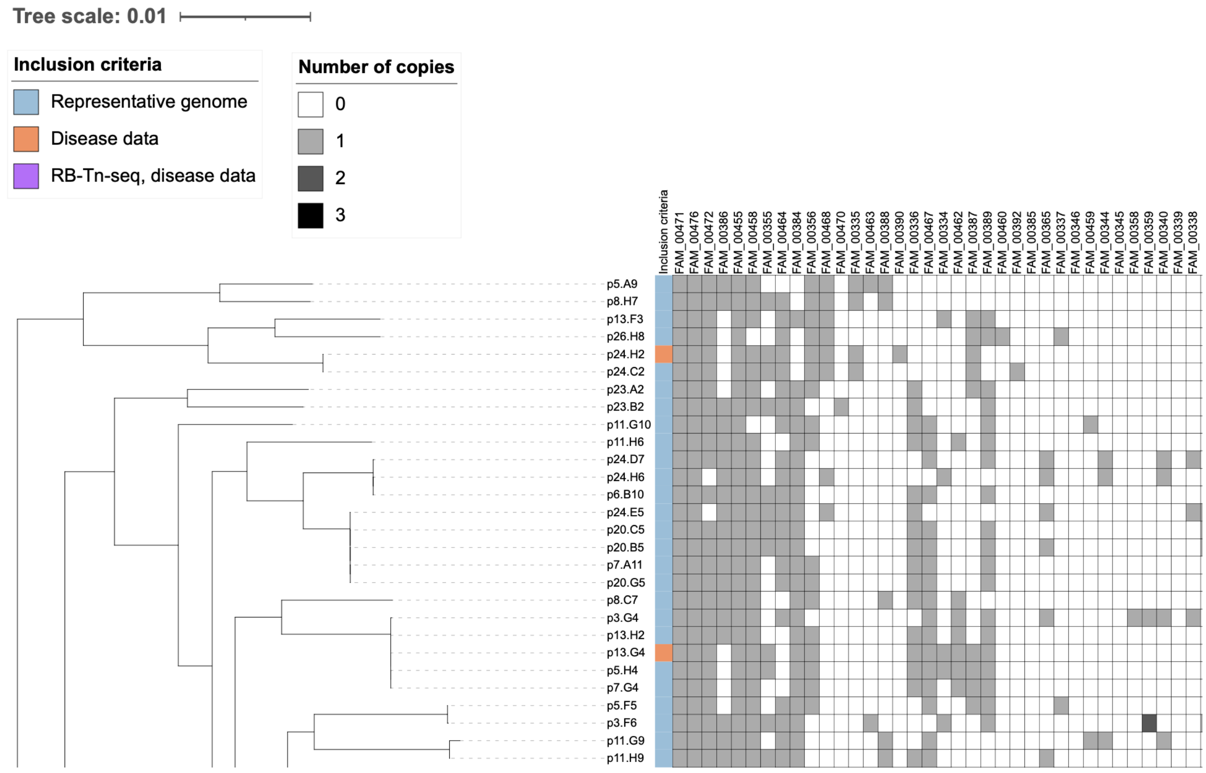

Q4: Following up on Q3: For the examples mentioned above (and the additional examples visible in the figure) it appears that there is a loss of correlation between BGC content and genome phylogeny. Does this influence your examination (and conclusions) of the correlation between "BGC" presence and disease?

Indeed, BGCs, defense mechanisms and other traits encoded in mobile elements can be uncoupled from other genomics features (8, 9). However, we believe our examination and conclusion stand. On the one hand, our RB-Tn-Seq analysis is based on one bacterial isolate infecting two host genotypes, so the loss of correlation between BGC content and bacterial phylogeny is not relevant here. On the other hand, we already included the phylogenetic relationship between *P. viridiflava* isolates via the treeWAS analysis, which accounts for population structure. Although we did not find significant BGCs here, the top GCFs candidates associated with disease severity identified by treeWAS were consistent with those identified by correlation analysis (Fig. 4D).

Q5: I suggest to use a label in Figure 1 so that both the representative strains and the "disease" strains are identifiable. Perhaps also the p25.C2 strain.

Thank you for this simple yet valuable suggestion. We have added the suggested label to Fig. 1, improving the figure's readability and interpretation.

Comment #2: Assessments of the fitness benefits/costs of BGC

Q6: There are examples of regions where some of the mutants have a positive fitness impact while others have a negative - For example regions 5 and 6 in Ey15-2 (figure 3ab). Can you speculate how this can happen?

In regions 5 and 6 (in Ey15-2) and region 7 (in Col-0), we identified DAMs with both positive and negative fold changes, i.e. indicating both fitness advantages and costs of the same BGC. There are several possible explanations for this. We assume —as is standard in Tn-seq studies— that a transposon insertion in any location of any of the genes of the BGC results in the knock-out of the gene cluster and the specialized metabolite not being produced. However, we did not confirm this experimentally. It could be that, in reality, insertions in different genes or different locations of a gene have a different impact on the

production, regulation and/or transport of the BGC product, which could explain having mutants with both positive and negative fitness impact in a single BGC. Furthermore, it could be that not all genes predicted to be part of a BGC by antiSMASH are indeed required for the specialized metabolite production and/or function (as already pointed out by the reviewer in Q8). antiSMASH developers indicate that their methods tend to overpredict BGC genes by over-extending the BGC, i.e., including adjacent genes that are not relevant for the specialized metabolite production (12). Finally, genes with opposite functions within the BGC could have different fitness effects without this being a contradiction, for example, genes encoding activators vs repressors.

We have added these points to the discussion and emphasized the need for further experimental validation of our results (lines 470, 497-504).

Q7: I think the reader may be interested in knowing which particular mutants had neutral, negative or positive effects. In other words, having access to the details which is plotted in Figure 3. Perhaps in an excel sheet. This information may become relevant for others that may work on these regions in the future.

We have included these data as tables S4 and S5.

Q8: I would argue that it is important to differentiate more clearly between "BGCs" and "BGC-associated genes" in your text (including in title and abstract). As I read your results, your analysis of the 11 BGC show that "only" one of them (region 6) contribute to fitness (and only in Ey15-2) while the remaining 10 do not (Table 2)! On the other hand, you do indeed find that about 10% of the total number of genes that are "associated" with (within) these 11 BGC do have fitness effects. But for many (if not most) of these genes it is unclear if they have any role in the "function" of the BGC. The boundaries and length of the BGC are somewhat arbitrary defined and the genes included in these clusters may in fact have nothing to do with the production of secondary metabolites. So I suggest to use "BGC-associated genes". In short: I am not convinced that finding one or more fitness-modulating 'BGC-associated genes' in most of the 11 BGCs is sufficient to conclude that the majority of BGCs affect fitness. Please clarify

We agree with the reviewer that the length and boundaries of a predicted BGC, which defines which genes are included, are somewhat arbitrary; nonetheless, a threshold has to be set to allow predictions and comparisons. The tool we used to predict BGC, antiSMASH, is the gold standard for prediction of biosynthetic gene clusters from bacterial genomes. antiSMASH uses manually curated and validated 'rules' to identify a BGC. After identifying the core biosynthetic genes that meet these rules, the BGC is extended both upstream and downstream of the core biosynthetic genes by an empirically determined extension value, which is specific for each BGC class. antiSMASH developers note that the extension values generally tend to overpredict BGC length (12), resulting in the inclusion of adjacent genes in a BGC that do not actually contribute to the production of its specialized metabolite.

A limitation of our study, as we have noted already, is that we did not experimentally check for the production of the specialized metabolites encoded by *P. viridiflava* nor for the effect on the specialized metabolite production of the random transposon insertions. It could well be that genes included in the BGC by antiSMASH are actually not relevant for secondary metabolite production and fitness *in planta*, thus confounding our conclusions.

We have mentioned this caveat explicitly in the discussion (lines 497-501, in conjunction to the answer to Q6) and changed "BGC genes" for the more nuanced "BGC-associated genes" wherever possible throughout the manuscript.

References

1. Karasov TL, Neumann M, Leventhal L, Symeonidi E, Shirsekar G, Hawks A, Monroe G, Pathodopsis Team, Exposito-Alonso M, Bergelson J, Weigel D, Schwab R. 2024. Continental-scale associations of Arabidopsis thaliana phyllosphere members with host genotype and drought. Nat Microbiol 9:2748–2758.

2. Parks DH, Chaumeil P-A, Mussig AJ, Rinke C, Chuvochina M, Hugenholtz P. 2026. GTDB release 10: a complete and systematic taxonomy for 715 230 bacterial and 17 245 archaeal genomes. Nucleic Acids Res 54:D743–D754.

3. Helmann TC, Deutschbauer AM, Lindow SE. 2019. Genome-wide identification of Pseudomonas syringae genes required for fitness during colonization of the leaf

surface and apoplast. Proc Natl Acad Sci U S A 116:18900–18910.

4. Helmann TC, Deutschbauer AM, Lindow SE. 2020. Distinctiveness of genes contributing to growth of Pseudomonas syringae in diverse host plant species. PLoS One 15:e0239998.

5. Brereton RG. 2021. Empirical and statistical *p* values and Type 1 error rates: Putting it all together. J Chemom 35:e3330.

6. Karasov TL, Almario J, Friedemann C, Ding W, Giolai M, Heavens D, Kersten S, Lundberg DS, Neumann M, Regalado J, Neher RA, Kemen E, Weigel D. 2018. Arabidopsis thaliana and Pseudomonas Pathogens Exhibit Stable Associations over Evolutionary Timescales. Cell Host Microbe 24:168–179.e4.

7. Shi Y-M, Hirschmann M, Shi Y-N, Ahmed S, Abebew D, Tobias NJ, Grün P, Crames JJ, Pöschel L, Kuttenlochner W, Richter C, Herrmann J, Müller R, Thanwisai A, Pidot SJ, Stinear TP, Groll M, Kim Y, Bode HB. 2022. Global analysis of biosynthetic gene clusters reveals conserved and unique natural products in entomopathogenic nematode-symbiotic bacteria. Nat Chem 14:701–712.

8. Hussain FA, Dubert J, Elsherbini J, Murphy M, VanInsberghe D, Arevalo P, Kauffman K, Rodino-Janeiro BK, Gavin H, Gomez A, Lopatina A, Le Roux F, Polz MF. 2021. Rapid evolutionary turnover of mobile genetic elements drives bacterial resistance to phages. Science 374:488–492.

9. Al-Siyabi N, Al-Ansari A, Abri MAA, Sivakumar N. 2025. Genomic insights into biosynthetic gene cluster diversity and structural variability in marine bacteria. Sci Rep 15:37644.

10. Ashkenazy H, Weigel D. 2025. PanGene-O-Meter: Intra-species diversity based on gene-content. bioRxiv.

11. Navarro-Muñoz JC, Selem-Mojica N, Mullowney MW, Kautsar SA, Tryon JH, Parkinson EI, De Los Santos ELC, Yeong M, Cruz-Morales P, Abubucker S, Roeters A, Lokhorst W, Fernandez-Guerra A, Cappelini LTD, Goering AW, Thomson RJ, Metcalf WW, Kelleher NL, Barona-Gomez F, Medema MH. 2020. A computational framework to explore large-scale biosynthetic diversity. Nat Chem Biol 16:60–68.

12. Blin K, Kim HU, Medema MH, Weber T. 2017. Recent development of antiSMASH and other computational approaches to mine secondary metabolite biosynthetic gene clusters. Brief Bioinform https://doi.org/10.1093/bib/bbx146.

Re: mSystems00212-26R1 (**Biosynthetic gene clusters in *Pseudomonas viridiflava* have a fitness cost during *Arabidopsis thaliana* infection**)

Dear Prof. Talia L Karasov:

Your manuscript has been accepted, and I am forwarding it to the ASM production staff for publication. Your paper will first be checked to make sure all elements meet the technical requirements. ASM staff will contact you if anything needs to be revised before copyediting and production can begin. Otherwise, you will be notified when your proofs are ready to be viewed.

Data Availability: ASM policy requires that data be available to the public upon online posting of the article, so please verify all links to sequence records, if present, and make sure that each number retrieves the full record of the data. If a new accession number is not linked or a link is broken, provide production staff with the correct URL for the record. If the accession numbers for new data are not publicly accessible before the expected online posting of the article, publication may be delayed; please contact ASM production staff immediately with the expected release date.

Publication Fees: For information on publication fees and which article types have charges, please visit our website. We have partnered with Copyright Clearance Center (CCC) to collect author charges. If fees apply to your paper, you will receive a message from no-reply@copyright.com with further instructions. For questions related to paying charges through RightsLink, please contact CCC at ASM_Support@copyright.com or toll free at +1-877-622-5543. CCC makes every attempt to respond to all emails within 24 hours.

ASM Membership: Corresponding authors may join or renew ASM membership to obtain discounts on publication fees. Need to upgrade your membership level? Please contact Customer Service at Service@asmusa.org.

Embargo Policy: A press release may be issued as soon as the manuscript is posted on the mSystems Latest Articles webpage. The corresponding author will receive an email with the subject line "ASM Journals Author Services Notification" when the article is available online.

Cover Image Submissions: If you would like to submit a potential Cover Image, please email a file and a short legend to msystems@asmusa.org. Please note that we can only consider images that (i) the authors created or own and (ii) have not been previously published. By submitting, you agree that the image can be used under the same terms as the published article. Image File requirements: TIF/EPS, 7.5 inches wide by 8.25 inches tall (at least 2,250 pixels wide by 2,475 pixels tall), minimum 300 dpi resolution (600 dpi preferred), RGB, and no figure elements, e.g., arrows or panel labels. The legend should be a short description of the image, 1-2 sentences recommended. Please download and use this interactive template in Adobe to ensure that your proposed cover image meets our size requirements (https://journals.asm.org/pb-assets/pdf-text-excel-files/ASM-Interactive-Sizing-Cover-Template-1715689791.pdf).

Thank you for submitting your paper to mSystems.

Sincerely,
Ákos T. Kovács
Editor
mSystems

Reviewer #1 (Comments for the Author):

I appreciate the author's thorough consideration of the questions and the subsequent revisions. I am satisfied with the changes and have no further questions.

Reviewer #2 (Comments for the Author):

The revised manuscript addresses all comments satisfactorily
